# Liquid solution centrifugation for safe, scalable, and efficient isotope separation

**DOI:** 10.1126/sciadv.adg8993

**Published:** 2023-07-12

**Authors:** Joseph F. Wild, Heng Chen, Keyue Liang, Jiayu Liu, Stephen E. Cox, Alex N. Halliday, Yuan Yang

**Affiliations:** ^1^ Department of Applied Physics and Applied Mathematics, Columbia University, New York, NY 10027, USA.; ^2^ Lamont-Doherty Earth Observatory, Columbia University, Palisades, NY 10964, USA.

## Abstract

A general method of separating isotopes by centrifuging dissolved chemical compounds in a liquid is introduced. This technique can be applied to almost all elements and leads to large separation factors. The method has been demonstrated in several isotopic systems including Ca, Mo, O, and Li with single-stage selectivities of 1.046 to 1.067 per neutron mass difference (e.g., 1.43 in ^40^Ca/^48^Ca), which are beyond the capabilities of various conventional methods. Equations are derived to model the process, and the results agree with those of the experiments. The scalability of the technique has been demonstrated by a three-stage enrichment of ^48^Ca with a total ^40^Ca/^48^Ca selectivity of 2.43, and the scalability is more broadly supported through analogies to gas centrifuge, whereby countercurrent centrifugation can further multiply the separation factor by 5 to 10 times per stage in a continuous process. Optimal centrifuge conditions and solutions can achieve both high-throughput and highly efficient isotope separation.

## Teaser

Centrifuging a liquid solution can efficiently separate element’s isotopes in both the solvent and the dissolved solutes.

## INTRODUCTION

The discovery of isotopes in the early 20th century led to countless world-changing technologies and applications. Enriched stable isotopes remain essential to help solve many of the most challenging questions in sustainability and fundamental science, such as ^6^Li as the source for generating ^3^H in nuclear fusion, ^48^Ca as a key source for producing superheavy elements and examining the Standard Model, and ^100^Mo as a precursor for ^99m^Tc within the broad field of radiopharmaceuticals (*1*).

Various methods have been developed for efficient isotope enrichment, including gas centrifuge, electromagnetism, gas diffusion, chemical exchange, and laser separation (*2*–*7*). Each method has its own advantages and disadvantages. For example, gas centrifuges are very successful at separating isotopes that can form gaseous molecules at near-ambient temperatures, such as UF_6_ for ^235^U/^238^U and Ni(PF_3_)_4_ for ^62^Ni/^64^Ni (*8*), but they are not suitable for isotopes that cannot be gasified at these temperatures, such as group I and group II elements. Moreover, most gaseous precursors are highly toxic. Electromagnetic isotope separation (EMIS) has near-perfect selectivity, but the production rate is extremely low and the cost is prohibitively high, making it only suitable for isotopes with a milligram–to–gram per year demand. Chemical methods use the isotope effects in the Gibbs free energy (e.g., molecular and atomic vibrations), which has been successful for light isotopes particularly with a large (1/M_1_ − 1/M_2_) such as hydrogen/deuterium (H/D) and ^6^Li/^7^Li. However, hazardous chemicals are often involved, such as H_2_S for H/D and Hg for ^6^Li/^7^Li, and the effect weakens substantially for heavier elements (*9*). Therefore, new methods of isotope enrichment with not only high selectivity and throughput but also less harmful environmental effects are desirable.

**Fig. 1.**
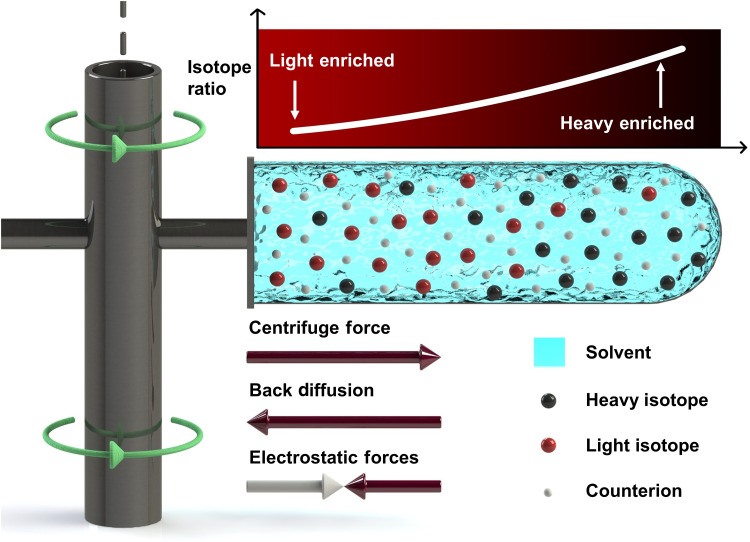
Schematic of the liquid solution centrifugation process. Heavier isotopes become more concentrated at the outer edge of the centrifuge, while lighter isotopes become more concentrated at the inner edge. The electrostatic force acts to attract the target ions and counterions to one another, resulting in charge neutrality. This schematic is to show the design of a swing bucket centrifuge used to produce experimental data in this work.

Here, we report a simple and universal liquid centrifuge method to separate isotopes for almost all elements, which is at or near-ambient temperature and pressure and does not involve hazardous materials. In general, a chemical containing the element requiring isotope separation (e.g., a salt) is dissolved in a solvent (e.g., water). The solution is then centrifuged, and the heavier isotopes are enriched at the outermost portions by centrifugal force, whereas the lighter isotopes are enriched at the innermost edge (Fig. 1). The process minimizes total free energy by forming a density gradient.

## RESULTS

### Modeling of liquid centrifuge

The centrifuge process is described by Eq. 1 and explained further in sections S2 and S3 of the Supplementary Materials

$$J_{i}=-D_{i}\vartheta \frac{\partial c_{i}}{\partial r}+D_{i}\frac{\omega^{2}r}{RT}c_{i}M_{i}(1-{\bar{v}}_{i}\rho_{\mathrm{soln}})+D_{i}c_{i}\frac{z_{i}FE}{RT}\;\mathrm{with}\;\vartheta =1+c\frac{\partial ln(\gamma )}{\partial c}$$ (1)

where *J* is the species flux, and *i* indexes an ionic isotope. *D* is the species diffusivity and ϑ is the thermodynamic factor of the Onsager-Fuoss model, which relates the diffusivity, *D_F_*, to the purely kinetic diffusivity *D_i_* through *D_F_* = ϑ*D_i_* (*10*). *c* is the molar concentration, *r* is the radius, ω is the angular velocity, *M* is the molar mass, *R* is the gas constant, and *T* temperature. $\bar{v}$ is the partial specific volume, and ρ_soln_ is the density of the solution. *z* is the valence of the ionic species, *F* is the Faraday constant, and *E* is the electric field. γ is the activity coefficient. At equilibrium, the diffusion flux due to the concentration gradient balances the mass-dependent flux arising from centrifugation and the electrostatic flux.

**Fig. 2.**
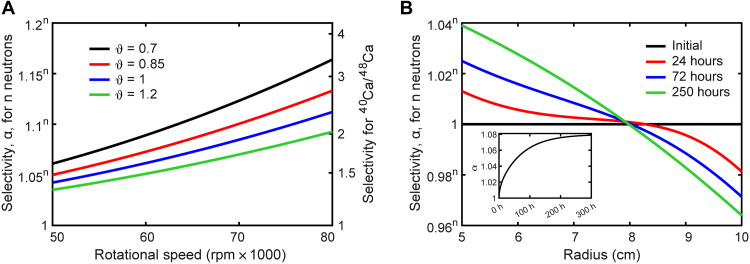
Simulation results of isotope separation in liquid solutions. (**A**) Equilibrium separation factors versus revolutions per minute for a rotor with *r*_i_ = 50 mm, *r*_o_ = 100 mm, and at 298 K. Left axis for a given element where “n” designates the neutrons between chosen isotopes. Right axis for the case of ^40^Ca/^48^Ca. (**B**) Time-dependent isotope ratio versus location for 70 kRPM, 40°C, and a 1:1 monovalent salt used in the simulation. *r*_i_ = 50 mm, *r*_o_ = 100 mm, *D*_+_ = *D*_−_ = 10^−9^ m^2^ s^−1^, and ϑ = 1. The inset figure gives the total selectivity per neutron mass difference against time.

The selectivity, α, which is defined as α = ([*M*_1_]/[*M*_2_])_inner_/([*M*_1_]/[*M*_2_])_outer_ with *M*_1_ < *M*_2_, can be expressed in equilibrium as

$$\alpha =\exp\left(\frac{\omega^{2}(M_{2}-M_{1})(r_{o}^{2}-r_{i}^{2})}{2\vartheta RT}\right)$$ (2)

where *r*_o_ and *r*_i_ are the outer and inner centrifuge radii, respectively. The selectivity equation is essentially identical to the gas centrifuge case, with the only difference being ϑ to account for nonidealities in the liquid solution (*11*). This equation has been previously applied to explain isotope fractionations observed in solid and molten metals upon centrifugation at elevated temperatures of >200°C (*12*–*14*). As shown in Fig. 2A, α can reach 1.05 to 1.1 per neutron difference at equilibrium at a practical rotation speed (e.g., 50,000 to 100,000 rpm), which is equivalent to 1.48 to 2.14 for ^40^Ca^/48^Ca.

Compared to gas centrifuges, the liquid centrifuge has the following advantages. First, it is suitable for most elements, while the pool for gas centrifuges is limited. For example, most elements that are difficult to form gaseous species near-ambient conditions, such as all group I and group II elements and the lanthanides, are incompatible with the gas centrifuge. In contrast, every element can be made to have good water solubility, except for the noble gases. Second, the isotope concentration in a liquid solution can be much higher than a gas. For instance, 1 M isotope solution is 22.4 times as concentrated as a gas at standard conditions, thus increasing throughput. Many elements can be dissolved with a concentration up to 5 to 10 M via a nitrate, nitrite, or halide (*15*). Third, ϑ is a factor that tends to be below 1 for most aqueous solutions at low concentrations and can become as low as 0.2 to 0.3 for certain salt/solvent combinations, particularly multivalent ions and low dielectric constant solvents (*16*), while ϑ is exactly 1 in an ideal gas (section S2.1). In principle, this could allow for the separation factor to be multiple times greater than a gas centrifuge with the same experimental parameters, albeit at low concentrations. Last, nonreactive solids and liquid solutions are much safer to handle than toxic gases (e.g., UF_6_), which have unfortunately led to fatal accidents (*17*).

### Enriching ^48^Ca by liquid centrifuge

Here, we focus on enriching ^48^Ca to demonstrate the capability of liquid centrifugation and use ^100^Mo and ^6^Li for further validation to represent broad classes of elements across the periodic table. ^48^Ca has a natural abundance of 0.187%, while ^40^Ca accounts for 96.941% of all Ca isotopes. Calcium has no suitable compound that can be gasified near-ambient temperature and is currently produced by EMIS with a low rate of ~10 g/year and price exceeding $100,000/g. ^100^Mo has a natural abundance of 9.74% and has important radiopharmaceutical applications (*18*). The current production method of ^100^Mo is either low throughput (EMIS) or involves toxic chemicals such as MoF_6_ in gas centrifugation. ^6^Li has a natural abundance of 7.5%, and its historical enrichment used more than 2 tons of toxic Hg to obtain every 1 kg of enriched ^6^Li via the column exchange (CoLEX) process (*19*). Here, we achieved a high selectivity of 1.434 for ^40^Ca/^48^Ca at 60 kilo revolutions per round (kRPM) with a commercial biomedical centrifuge after 72 hours, while literature has only reported 1.005 to 1.012 in chemical separation (*20*, *21*) and 1.26 in a 14-day-long thermal diffusion (*22*). Similarly, a selectivity of 1.054 was realized in ^6^Li/^7^Li, essentially identical to the COLEX process selectivity, while 1.485 was achieved in ^92^Mo/^100^Mo.

To enrich ^48^Ca by liquid centrifugation, CaCl_2_, Ca(NO_3_)_2_, and CaS_2_O_3_ were dissolved in water to form 0.1, 1, 2, or 5 mol kg^−1^ solutions, which were centrifuged in a tube for 24 or 72 hours at 40°C. Samples were then taken from the top and bottom of the tubes and were analyzed with an Nu Instruments Sapphire collision cell–equipped multicollector inductively coupled plasma mass spectrometry (MC-ICPMS) with an ultrahigh accuracy corresponding to <±0.00065 selectivity measurement error (*23*, *24*). The top and bottom of the tubes correspond to the inner and outer radii, respectively.

**Fig. 3.**
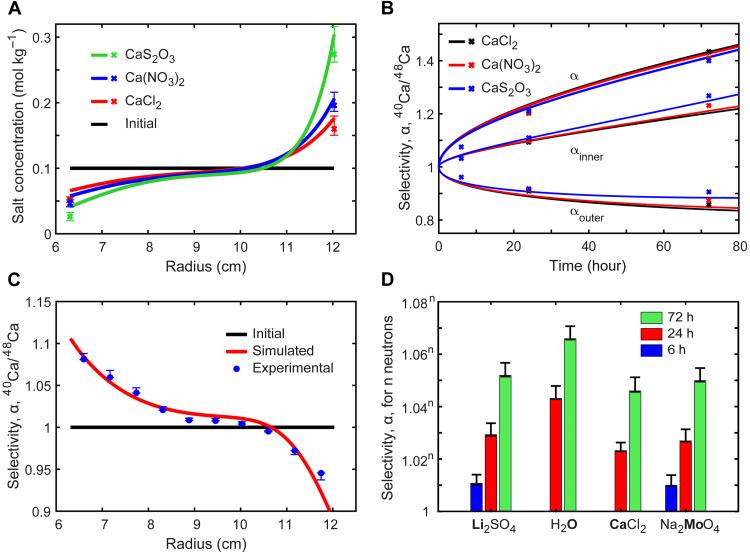
Results of isotope separations by liquid centrifuge. (**A**) Simulated concentration distributions (lines) for three calcium salts after 24 hours along with experimental values (crosses) at the inner and outer radii. (**B**) Simulated and experimental separation factors versus time for three Ca salts at 0.1 mol kg^−1^. (**C**) Simulated and experimental spatial distribution of ^40^Ca/^48^Ca selectivity in Ca(NO_3_)_2_ after 24 hours. (**D**) Measured separation factor after 6, 24, and 72 hours in selected bold elements at 0.1 mol kg^−1^ in H_2_O. Variations between elements are primarily caused by their different (ionic) diffusivities. The results are normalized to the neutron difference, n.

First, all salts tend to concentrate at the outer radii because they are denser than water, and the results are consistent with modeling predictions (Fig. 3A and tables S13 and S14). Relatively flat concentration gradients at ≥5 mol kg^−1^ likely result from the theoretically understood nonlinearities of transport of concentrated electrolytes. The different degrees of concentration polarization mainly originate from the magnitude of *M*_salt_(1 − $\bar{v}$_salt_ ρ_soln_), which represents the centrifugal driving force to induce these polarizations. The different diffusivities and ϑ also affect the result but to a lesser magnitude. For example, a higher concentration of salt leads to smaller polarization due to a reduced diffusivity.

As with concentration polarizations, heavier ^48^Ca also concentrates at the outer radii compared to ^40^Ca (Fig. 3B). Upon time, α increases from 1.202/1.201/1.210 at 24 hours to 1.434/1.410/1.400 at 72 hours for 0.1 M CaCl_2_, Ca(NO_3_)_2_, and CaS_2_O_3_, respectively, which is consistent with the model prediction that the counterion does not substantially affect α. The uncertainty of α is (+0.028, −0.0003) for 6 hours, (+0.035, −0.0003) for 24 hours, and (+0.037, −0.0003) for 72 hours (see section S5.1 for further details). α generally decreases with increasing concentration as the reduced diffusivity slows down the separation before reaching equilibrium. For example, in CaCl_2_, α decreases from 1.202/1.434 at 0.1 M to 1.162/1.352 at 2 M to 1.106/1.194 at 5 M for 24 hours/72 hours, respectively (fig. S2). However, as the enriched isotope flux is proportional to salt concentration, a higher concentration often favors a larger throughput for practical applications. The diffusivity can also be enhanced by increasing temperature, as it increases by ~2.5%/K in aqueous solution, although this will slightly decrease the equilibrium selectivity due to the *T*^−1^ dependence of Eq. 2 (*25*).

Along with the separation factor, the symmetry of isotope enrichment at the two ends of the centrifuge relative to the initial isotope ratio also plays an important role in the separation. With the same α, if enrichment of the heavier isotope is targeted (e.g., ^48^Ca), a lower ([*M*_1_]/[*M*_2_])_outer_/([*M*_1_]/[*M*_2_])_initial_ (α_outer_) is preferred if *M*_1_ < *M*_2_. If the target isotope is the lighter one (e.g., ^10^B), then a higher ([*M*_1_]/[*M*_2_])_inner_/([*M*_1_]/[*M*_2_])_initial_ (α_inner_) is preferred. As shown in Fig. 3B, α_outer_ and α_inner_ are approximately symmetric within 24 hours, but with increasing time, α_outer_ starts to saturate, while α_inner_ keeps increasing. CaCl_2_ tends to have the highest α_outer_, and it was found that this can be mainly attributed to the value of *M*_salt_(1 − $\bar{v}$_salt_ ρ_soln_), with a smaller value favoring a higher proportion of enrichment at the outer radii.

Ideally, a large proportion of the solution would have high isotopic enrichment. To determine the spatial distribution, 10 wt % gelatin was added in the aqueous solution, and the temperature was decreased to 0°C for the last 3 hours of centrifugation. The solution would mostly gelatinize, and the spatial distribution could be determined without being disturbed by convection. It is found that the heavy isotope is enriched at the bottom ~1/4 of the tube, which is consistent with model predictions for the calcium nitrate salt (Fig. 3C). However, as the salt is more concentrated at the bottom, ~1/3 of the total salt is enriched with ^48^Ca, and ^40^Ca/^48^Ca is roughly linear with atomic percentage in the enriched region.

### Generalization to other isotopes

To further show that the liquid centrifuge method is universal across the periodic table, we applied the same method to ^6^Li/^7^Li, ^39^K/^41^K, and all seven Mo isotopes. In many cases, the same salt was used to separate the isotopes of both the anion and cation, e.g., Li_2_MoO_4_. As shown in Fig. 3D, Li_2_SO_4_ gives an α of 1.052 for ^6^Li/^7^Li at 72 hours, while Na_2_MoO_4_ gives an α of 1.485 for ^92^Mo/^100^Mo, which corresponds to 1.0507 per neutron mass difference. These data confirm that α scales with Δ*M* and demonstrates the universality of the liquid centrifuge method. This is further confirmed by comparing α among different isotope pairs in molybdenum (section S4). Moreover, by analyzing ^1^H/^2^H and ^16^O/^18^O in the solvent, separation factors of 1.067 and 1.134 were found, respectively, thereby indicating that isotopes within the solvent itself were effectively separated. The high self-diffusivity of water and its isotopologs means that 72 hours is sufficient to closely approach equilibrium, thereby explaining the higher value per neutron mass difference of 1.065 to 1.067 compared to that for dissolved ions.

## DISCUSSIONS

### Cascading and scalability

Continuous centrifugation is important for the practical applications of this strategy, which is currently under investigation and will be reported in the future. Here, to demonstrate the simple scalability of liquid solution centrifugation, we performed a three-stage enrichment of ^48^Ca. Each solution was centrifuged for 72 hours and then the top and bottom 10% of the solution was collected. These solutions were then diluted and centrifuged for an additional 72 hours, and the process was repeated. The dilution step would not be required in a continuous process because the exit stream of one stage would directly feed into another. The results of these enrichments are shown in table S15, with the ^48^Ca abundance reaching 0.282 and 0.116 atomic % (at %) after the three-stage enrichment compared to its natural abundance of 0.187 at %. This corresponds to a separation factor of 2.43 after three stages or equivalently 2.43^1/3^ = 1.34. Given that we use the top and bottom 10% volume in this three-stage experiment instead of the very top and bottom in a single-stage experiment, these results are consistent with a separation factor of ~1.40 in a single stage.

The scalability of liquid centrifugation to a continuous process is supported by analogy with the widely used gas centrifuge method and associated countercurrent devices. In this case, the feed and product streams are continuous, while the centrifuge continues to rotate at the target speed. Internal flow profiles are induced that lead to much larger separation factors along the axial direction than can be achieved in the radial direction alone. This has the effect of multiplying the single-stage separation factor by many times depending on the height/diameter ratio, such that even under modest centrifuging conditions, selectivities exceeding 1.20 per neutron difference can be achieved (e.g., >4.30 in ^40^Ca/^48^Ca and >1.44 in ^16^O/^18^O). This countercurrent centrifugation method greatly simplifies the cascading process by reducing the number of stages and has successfully led to the enrichment of 1000’s of tons of ^235^U. Potential possible methods to apply countercurrent flow include a thermal gradient, conical/tapered rotors, or by the mechanism of feed introduction and product/waste removal (*11*). However, it should be cautioned that these methods’ feasibility requires careful examinations in the future because liquids’ fluid properties are very different from gases. For example, the mass diffusivity in liquid is significantly lower than gas, and the viscosity in liquid is much higher than gas. This is the focus of the authors’ current studies and will be reported in the future. These principles are further discussed in section S7.

One potential challenge for the technique is that the diffusivity of ions in water is typically 1 × 10^−9^ to 2 × 10^−9^ m^2^ s^−1^ at 25°C, which is about three orders of magnitude less than gases for centrifugation (~10^−6^ m^2^ s^−1^; table S18). At a steady state, the isotope flux is proportional to diffusivity. However, this can be largely compensated by the higher concentration of isotopes in a liquid, which is commonly >10^2^ times that of a gas for dissolved salts and 10^3^ to 10^4^ times for solvent isotopes because the isotope flux is also proportional to its concentration (section S8). Moreover, steady-state fluxes can be increased with temperature because the diffusivity and, generally, the salt solubility increase significantly with temperature. This dependence is much stronger in a liquid than in a gas (fig. S4). Therefore, liquid centrifugation can achieve steady-state fluxes around ~1/10 that of gas centrifuges at ambient conditions, with elevated temperatures (60° to 100°C) allowing additional increases of two to three times.

Using the analogy of gas centrifuges and their available cost data for separative work, the production costs may be approximated for a liquid solution assuming similar power consumptions in both cases (*26*, *27*). The price per mole of separative work (MSW) is estimated as ($70 to $120 per Δ*M*) for a given element, where Δ*M* is the neutron difference between the isotopes of interest. This is in comparison to ~($71 per Δ*M*) per MSW for UF_6_ in operational gas centrifuges. Analysis indicates ~$15 per MSW for ^40^Ca/^48^Ca. In general, the variations between elements depend on the maximum isotope fluxes and Δ*M*, with larger values allowing for more efficient separation. It should be cautioned that the power needed for liquid centrifugation requires further examinations. Further details are provided in section S9.

### Cocentrifugation and solvent selection

The overall effectiveness and efficiency of this technique could be improved in real systems through several considerations, such as increasing the peripheral speed, using countercurrent centrifugation, increasing temperature, and adjusting *r*_i_ and *r*_o_. Last, the isotopes of multiple desired elements can be separated at the same time by using carefully chosen salts, such as Li_2_MoO_4_ or CaCl_2_ to simultaneously separate both Li and Mo or Ca and Cl, respectively. Moreover, isotopes in the solvent are separated and enriched simultaneously in the liquid centrifuge. Therefore, many isotopes can be enriched simultaneously such as ^2^H, ^18^O, ^37^Cl, and ^48^Ca, with H_2_O being highly concentrated at ~50 to 55 M and ^18^O important for producing ^18^F for positron emission tomography (*28*).

An additional factor to explore in liquid centrifugation is the solvent. On the basis of the Debye-Hückel theory, a solvent with lower dielectric constant (e.g., organic solvents) could reduce ϑ to 0.3 to 0.5 at low concentrations, which could increase the selectivity by 50 to 200% (*29*). However, these solvents often result in an ionic diffusivity one to two orders of magnitude lower than water, and so the transient selectivities in organic solvents in our preliminary studies were lower than in water (table S14). However, it remains a possibility that organic solvents with low viscosities and large nonidealities could provide better results than water in some cases, including cases where the target isotope is inside these solvents (section S10).

In general, at moderate centrifuge speeds and radii, new single-stage separation factor benchmarks could be set for most of the elements. This requires no adaptations to the centrifugation setup in any cases, and therefore, developments of the technique and hardware for a particular elemental system, e.g., improvements to the centrifuge materials, represent progress for all elements. This method has potential to improve the supply of hundreds of stable isotopes, which are used in various areas of energy generation, fundamental science, and radiopharmaceuticals, thereby aiding many of the important questions throughout fields of science.

## MATERIALS AND METHODS

### Preparing solutions

All chemicals used are listed in table S1. All solutions to be centrifuged were prepared in 10 g of deionized water (Direct-Q 3 UV water purification system). For example, LiCl (5 mol kg^−1^) was prepared by adding 0.05 mol (2.12 g) of anhydrous LiCl to 10 g of water. If the salt was initially hydrated, the mass of the water in the hydrated salt was subtracted from the 10 g of water. For example, CaCl_2_ (2 mol kg^−1^) was prepared by adding 0.02 mol (2.94 g) of CaCl_2_·2H_2_O to 9.28 g of water, because 0.72 g of water was already in the hydrated salt. Solutions were prepared in 22-ml polypropylene vials, which had been cleaned with deionized water and ethanol to avoid ion contamination from vials. Each centrifuge tube has a volume of around 4.0 ml. Two centrifuge tubes were used for each solution to check repeatability, and these were placed on opposite sides of the rotor after ensuring equal masses for stability.

### Centrifugation

The SW 60 Ti rotor in the Beckman Optima XPN-100 Ultracentrifuge was used at 60,000 rpm for all experiments. The inner and outer radii are 63.1 and 120.3 mm, respectively. It took 4 to 5 min to reach 60,000 or 0 rpm at the end of the run. The centrifuge automatically engaged its vacuum system when the rotor reached 3000 rpm. The rotor was generally initially at 15° to 20°C upon starting centrifugation, and the heating rate was found to be around 0.4° to 0.5°C min^−1^, so it would take around 1 hour to reach 40°C. At the end of the run, the temperature was set to 25°C for 1 hour at the same speed to bring the solution closer to ambient conditions and minimize convection-induced remixing upon collection. Open-top thin-wall polypropylene tubes were used in all experiments.

### Sample collection

Sterile needles (0.5 mm) were used to collect the samples from the top and bottom of the centrifuge tubes immediately after the end of the run. This process would take around 10 min for all six tubes. In general, 25 to 75 mg of the sample were collected in each case. The mass of the collected samples was measured by calculating the difference between the mass of the sample container before and after collection to 0.1 mg. This allowed for the concentration to be later determined. The top liquid could be accessed at the top of the centrifuge tube, while the bottom liquid was accessed by carefully removing the thin-wall tubes from the bucket and then slowly piercing the bottom of the tube in a twisting motion.

### Isotopic and concentration measurements

A Nu Sapphire MC-ICPMS (SP004, equipped with collision cell) was used for all K and Ca concentration and isotope measurements under collision cell mode (low-energy mode) to minimize spectral interference from the argon support gas fueling the plasma. A separate Nu Sapphire MC-ICPMS (SP005 without collision cell) was used for all Li and Mo measurements. A Picarro L2130i was used for the water H and O isotope measurements. The collected solutions, as well as the references, were diluted to 50 to 300 parts per billion in 2 wt % nitric acid. Once the concentration measurements had been made by comparing the intensities of the top and bottom solutions to the reference, the concentrations of all samples were brought within 5% of the standard solution concentration. Isotopic measurements were then made three times for each sample, and each analysis consists of 40 (50 for Li) cycles of 4-s (3 s for Li) integrations. Before isotopic measurements, the calcium samples were first passed through chromatography columns filled with Sr-Spec resin to remove interference element of Sr. The K samples were purified by chromatography columns filled with AG50-X8 cation exchange resin and measured following the method described by Chen *et al.* (*24*). Every isotope sample and the National Institute of Standards and Technology standard solution for that element were measured alternately (sample-standard bracketing) for mass bias and signal drift correction (*23*, *24*).

## Supplementary Materials

### This PDF file includes:

Materials and Methods Supplementary Text Figs. S1 to S4 Tables S1 to S20 References
