# Supplementary material for: Liquid solution centrifugation for safe, scalable, and efficient isotope separation
Source: Sci Adv. 2023 Jul 12;9(28):eadg8993. doi: 10.1126/sciadv.adg8993 (PMC10337892; doi:10.1126/sciadv.adg8993)
Supplement: Supplementary file 1 — Materials and Methods Supplementary Text Figs. S1 to S4 Tables S1 to S20 References [file sciadv.adg8993_sm.pdf]

Science Advances
AAAS

Supplementary Materials for

Liquid solution centrifugation for safe, scalable, and efficient isotope separation

Joseph F. Wild *et al.*

Corresponding author: Yuan Yang, yy2664@columbia.edu; Alex N. Halliday, alexhalliday@climate.columbia.edu

Sci. Adv. **9**, eadg8993 (2023)
DOI: 10.1126/sciadv.adg8993

This PDF file includes:

Materials and Methods
Supplementary Text
Figs. S1 to S4
Tables S1 to S20
References

Section S1. Materials used

Table S1 – Chemicals used and sources

Chemical	Source	Notes
calcium chloride dihydrate, ⩾ 99.0%	Sigma, C3306	Lot: SLBZ8395
calcium chromate, 99.9%	Alfa Aesar, 43333-14	Metals basis, Lot: R27H007
calcium nitrate tetrahydrate, 99%	Sigma, 237124	Lot: MKCQ1963
calcium thiosulfate, pure 30-50% solution in water	Acros Organics, 447870010	Liquid solution of density 1245 kg m^{-3} (~1.93 M), Lot: A0412159
dimethyl sulfoxide, 99.9% min	Alfa Aesar, 36480	Lot: X17C012
lithium bis(trifluoromethanesulfonyl)imide	Gotion	Stored in Ar glovebox
lithium bromide, anhydrous, ⩾ 99%	Sigma, 746479	Lot: MKCH6662
lithium chloride, anhydrous, 98+%	Alfa Aesar, A10531	Lot: 10189192, Stored in Ar glovebox
lithium hydroxide monohydrate, ⩾99.0%	Sigma, 62528	Lot: BCBZ5579
lithium molybdenum oxide, (lithium molybdate), 99+%)	Thermo Fisher, 13427	Lot: W16G020
lithium nitrate	Sigma, 227986	Lot: MKCK5638
lithium oxalate, 99+%	Thermo Fisher, 13426	Lot: R13I014
lithium sulfate, ⩾98.5%	Sigma, L6375	Lot: BCBP6592V
nitric acid, ICP-OES	Thermo Scientific, T00309-0500	Lot: 201922, For trace metal analysis
propylene carbonate, anhydrous, 99.7%	Sigma, 310328	Lot: SHBJ2151
sodium molybdate, anhydrous, 99.9% trace metal basis	Sigma, 737860	Lot: MKBH2923V
triethyl phosphate, ≥99.8%	Sigma, 538728	Lot: MKCJ1157, Stored in Ar glovebox

Section S2. Separation of Neutral Species in Equilibrium

2.1. Ideal Gases and Liquids: With the discovery of isotopes in the 1910's, Lindemann and Aston (*30*) first proposed centrifugation as a method of separation in 1919, and derived the following equilibrium governing equation for the case of an ideal gas or incompressible ideal liquid:

$$\alpha = exp\left(\frac{\omega^2(M_2 - M_1)(r_o^2 - r_i^2)}{2RT}\right)$$

2.2. Nonideality: In 1963, Hooyman (*31*) applied diffusion thermodynamics to centrifugation in multicomponent systems for the following equilibrium equation:

$$\frac{M_i\omega^2 r}{RT}[1 - \bar{v}_i(r)\rho(r)] = \frac{1}{c_i(r)}\frac{dc_i(r)}{dr} + \sum_{k=1}^{v-1}\left[\frac{\partial \ln(\gamma_i^{(c)})}{\partial c_k(r)}\right]_{T,P,c_{j\neq k}}\frac{\partial c_k(r)}{dr}$$

In the case of a dilute solution of chemically identical isotopic species, this equation becomes:

$$\frac{M_i\omega^2 r}{RT}[1 - \bar{v}_i\rho_0] = \frac{1}{c_i(r)}\left[1 + c_{tot}(r)\frac{\partial \ln(\gamma)}{\partial c_{tot}(r)}\right]\frac{dc_{tot}(r)}{dr} = \frac{\vartheta(c_{tot})}{c_i(r)}\frac{dc_i(r)}{dr}$$

If the concentration dependence of the thermodynamic factor can be neglected, then the resulting differential equation is separable and can be solved analytically:

$$\frac{M_i\omega^2 r}{RT}[1 - \bar{v}_i\rho_0]\,dr = \frac{\vartheta}{c_i(r)}dc_i(r) \quad \rightarrow \quad c_i(r) = B_i\,exp\left(\frac{\omega^2 M_i[1 - \bar{v}_i\rho_0]r^2}{2\vartheta RT}\right)$$

Then, if it is assumed that the partial molar volume of a chemical is the same for isotopes, $M_1\bar{v}_1 = M_2\bar{v}_2$. Finally, using the definition of the selectivity:

$$\alpha = \frac{c_2(r_o)/c_1(r_o)}{c_2(r_i)/c_1(r_i)} = \frac{\frac{B_2}{B_1}exp\left(\frac{\omega^2(M_2 - M_1){r_o}^2}{2\vartheta RT}\right)}{\frac{B_2}{B_1}exp\left(\frac{\omega^2(M_2 - M_1){r_i}^2}{2\vartheta RT}\right)} = exp\left(\frac{\omega^2(M_2 - M_1)(r_o^2 - r_i^2)}{2\vartheta RT}\right)$$

This equation neglects the pressure dependence of the thermodynamic factor as well as any solvation shell affects. As will be shown in Section 3, this final equation does not change if the anion and cation are treated separately, and their motion is coupled via an electric field term.

Section S3. Ionic Species Kinetics and Thermodynamics

3.1. Kinetic Model: Upon the dissolution of a salt into a solvent, the ions will tend to disassociate. Each ion will then respond to an external field depending on its own physical properties, i.e., ionic mobility, mass, volume, charge, etc. Macroscopically, the anion and cation move together due to the condition of charge neutrality, and so they are coupled via their electrostatic interaction. To incorporate this into a single model, the following equations were proposed for the 1D case. In the most simplified case of a single isotopic anion and two isotopic cations, they are:

$$J_- = -D_- \vartheta \frac{\partial c_-}{\partial r} + D_- \frac{\omega^2 r}{RT} c_- M_- (1 - \bar{v}_- \rho_{soln}) + D_- c_- \frac{z_- FE}{RT} \quad \text{(S1)}$$

$$J_{+,1} = -D_+ \vartheta \frac{\partial c_{+,1}}{\partial r} + D_+ \frac{\omega^2 r}{RT} c_{+,1} M_{+,1} (1 - \bar{v}_{+,1} \rho_{soln}) + D_+ c_{+,1} \frac{z_{+,1} FE}{RT} \quad \text{(S2)}$$

$$J_{+,2} = -D_+ \vartheta \frac{\partial c_{+,2}}{\partial r} + D_+ \frac{\omega^2 r}{RT} c_{+,2} M_{+,2} (1 - \bar{v}_{+,2} \rho_{soln}) + D_+ c_{+,2} \frac{z_{+,2} FE}{RT} \quad \text{(S3)}$$

The first term on the right is the Fickian flux, the second is the centrifugal flux, and the third term is the electrostatic flux. The electrostatic term is the only one that couples the ions together.

In addition to these, there is also the conservation of mass and the boundary/initial conditions:

$$\frac{\partial c_i}{\partial t} = -\frac{\partial J_i}{\partial r} \quad with \quad J_i(r_{inner}, t) = J_i(r_{outer}, t) = 0 \quad and \quad c_i(t = 0, r) = c_{o,i}$$

In general, these equations are:

$$\vec{J_i} = -D_i \vartheta \nabla c_i + D_i \frac{\omega^2 \vec{\boldsymbol{r}}}{RT} c_i M_i (1 - \bar{v}_i \rho_{soln}) + D_i c_i \frac{z_i F \vec{\boldsymbol{E}}}{RT} \quad with \quad \frac{\partial c_i}{\partial t} = -\nabla \cdot (\vec{J_i})$$

3.2. Solving Equations: The equations were solved numerically in MATLAB. In general, the governing flux equation can be discretized and solved for at each discretized spatial point at a given timestep. then Fick's second law be used to calculate the spatial distribution at the next timestep and so on.

The governing equation (eq. 1 in the main text) is

$$J_i = -D_i \vartheta \frac{\partial c_i}{\partial r} + D_i \frac{\omega^2 r}{RT} c_i M_i (1 - \bar{v}_i \rho_{soln}) + D_i c_i \frac{z_i FE}{RT} \quad with \quad \vartheta = 1 + c \frac{\partial ln(\gamma)}{\partial c}$$

The first approximation is to have $\vartheta = 1$, since electrolyte is nearly ideal at low concentration. We also only consider 1D cartesian coordinate for simplicity. This gives

$$J_i = J_{i,D} + J_{i,c} + J_{i,E} = -D_i \frac{\partial c_i}{\partial r} + D_i \frac{\omega^2 r}{RT} c_i M_i (1 - \bar{v}_i \rho_{soln}) + D_i c_i \frac{z_i FE}{RT}$$

Where $J_{i,D}$, $J_{i,c}$ and $J_{i,E}$ are fluxes due to chemical diffusion, centrifuge force and electrical field, respectively.

Step 1: Calculate $J_{i,D} + J_{i,c}$ at each location using the previous concentration distribution (for all r at a given t):

$$J_{i,D} + J_{i,c}(t + dt, r) = -D_{i,F}\frac{c_i(t, r + dr) - c_i(t, r - dr)}{2 \times dr} + D_i\frac{\omega^2 r}{RT}c_i(t, r)(M_i - \bar{M}_{bulk})$$

Step 2: Calculate $J_{i,E}$ and J_i. This is calculated with the assistance of charge quasi-neutrality in all locations and for all times, $\Sigma z_i c_i = 0$. For example, with one anion and two isotopic cations (e.g., Cl^-, ^{40}Ca and ^{48}Ca in $CaCl_2$), this equals to $z_- c_-(r, t) = -z_+(c_{+,1}(r, t) + c_{+,2}(r, t))$.

Such electroneutrality originates from the Poisson equation $\Delta\varphi = F\Sigma z_i c_i/\varepsilon$. Since $F/\varepsilon > 10^{14}$ V m mol^{-1} in water, even if $\Delta\varphi$ were of the order of 1 kV mm^{-2} (10^9 V m^{-2}), then $\Sigma z_i c_i < 10^{-5}$ mol m^{-3} = 10 nmol L^{-1}. Therefore, the solution can be treated as electrically neutral with the overall anion and cation charge balancing everywhere.

Mass conservation in 1D requires $\frac{\partial c_i}{\partial t} + \frac{\partial j_i}{\partial r} = 0$. Combining it with $\sum z_i c_i = 0$, we have $\frac{\partial}{\partial r}(\sum z_i j_i) = 0$.

As $j_i = 0$ for all species at the two ends at a centrifuge tube, this means

$$\sum z_i J_i(r, t) = 0$$

at all r (radii) and for all t (time). The physical picture that there is no movement of net charge as the electrolyte is electro-quasi-neutral.

As $J_i = J_{i,D} + J_{i,c} + J_{i,E}$, and $J_{i,D} + J_{i,c}$ are calculated in step 1, therefore, we have

$$\sum z_i J_{i,E}(r, t) = -\sum z_i(J_{i,D}(r, t) + J_{i,c}(r, t))$$

which becomes a known value. Then given that

$$J_{i,E}(r, t) = D_i c_i\frac{z_i F E(r, t)}{RT}$$

where E only depends on position and time, but not ions. This means that for a specific species k, its electrical flux $J_{k,E}(r, t)$ can be expressed as

$$J_{k,E}(r, t) = D_k c_k z_k\frac{-\sum z_i(J_{i,D}(r, t) + J_{i,c}(r, t))}{\sum_i D_i c_i z_i^2}$$

Then J_k is calculated as $J_k = J_{k,D} + J_{k,c} + J_{k,E}$,

Step 3: No flux at the inner and outer radii boundaries (r_i and r_o):

$$J_i(t, r_i) = 0 \quad and \quad J_i(t, r_o) = 0$$

Step 4: With Fick's second law, calculate the new concentration at each location using the fluxes in and out of the discretized element:

$$c_i(t + dt, r) = c_i(t, r) - dt \times \frac{J_i(t, r + dr) - J_i(t, r - dr)}{2 \times dr}$$

Step 5: Concentration changes at boundaries:

$$c_i(t+dt, r_{i/o}) = c_i(t, r_{i/o}) \mp dt \times \frac{J_i(t, r \pm dr)}{2 \times dr}$$

Go back to Step 1 and repeat for all t.

By repeating procedures above, the time dependent concentration distribution of each species can be determined. This method was used to create all simulated distributions labelled in figures.

3.3. Equilibrium Derivation: The equilibrium selectivity can be derived from the above equations with the electrostatic term as follows, which reveals the same equation as at the end of Section S2. This value was also converged upon as $t \to \infty$ in the MATLAB simulation, as shown in Fig. 2b:

At equilibrium, $J_{+,1} = J_{+,2} = 0$. Then (S3) × $c_{+,1}$ - (S2) × $c_{+,2}$ leads to

$$\vartheta c_{+,1} \frac{\partial c_{+,2}}{\partial r} - \vartheta c_{+,2} \frac{\partial c_{+,1}}{\partial r} = \frac{\omega^2 r}{RT} c_{+,1} c_{+,2} \left[M_{+,2}\left(1 - \bar{v}_{+,2}\rho_{soln}\right) - M_{+,1}\left(1 - \bar{v}_{+,1}\rho_{soln}\right) \right]$$

Then using $M_{+,1}\bar{v}_{+,1} = M_{+,2}\bar{v}_{+,2}$ and the reverse quotient rule:

$$\frac{\partial \left(\frac{c_{+,2}}{c_{+,1}}\right)}{\partial r} = \frac{\omega^2 r}{\vartheta RT} \frac{c_{+,2}}{c_{+,1}} \left(M_{+,2} - M_{+,1}\right)$$

This is then separable in r and $\frac{c_{+,2}}{c_{+,1}}$:

$$\frac{c_{+,1}}{c_{+,2}} d\left(\frac{c_{+,2}}{c_{+,1}}\right) = \frac{\omega^2 r}{\vartheta RT}\left(M_{+,2} - M_{+,1}\right) dr \quad \rightarrow \quad \frac{c_{+,2}}{c_{+,1}}(r) = B_o exp\left(\frac{\omega^2 (M_2 - M_1) r^2}{2\vartheta RT}\right)$$

Finally, using the definition of the selectivity:

$$\alpha = \frac{\frac{c_{+,2}}{c_{+,1}}(r_o)}{\frac{c_{+,2}}{c_{+,1}}(r_i)} = \frac{B_o exp\left(\frac{\omega^2 (M_2 - M_1) {r_o}^2}{2\vartheta RT}\right)}{B_o exp\left(\frac{\omega^2 (M_2 - M_1) {r_i}^2}{2\vartheta RT}\right)} = exp\left(\frac{\omega^2 (M_2 - M_1)\left(r_o^2 - r_i^2\right)}{2\vartheta RT}\right)$$

Therefore, the same equilibrium selectivity is obtained whether or not the electrostatic interaction is considered.

Section S4. Mass Dependence

4.1. Choice of Element: To verify that the natural logarithm of the selectivity among different isotopes is strictly proportional to their mass difference, even at the transient state, the selectivities were measured among different isotope pairs of an element after 72 hours of liquid centrifugation. Molybdenum was chosen since this element has the highest number of highly abundant stable isotopes - all seven of its stable isotopes have high natural abundances between 9.19% and 24.29% and can therefore be measured to high precision. Moreover, molybdenum isotopes only interfere directly with some zirconium and ruthenium isotopes which do not tend to naturally contaminate molybdenum sources, and therefore background interferences can be effectively removed. The isotopes of molybdenum, along with their masses and natural abundances (at%), are given in Table S2.

Table S2 – Masses and abundances of Mo isotopes

Isotope	Mass (Da)	Natural Abundance
^{92}Mo	91.90681	14.65%
^{94}Mo	93.90509	9.19%
^{95}Mo	94.90584	15.87%
^{96}Mo	95.90468	16.67%
^{97}Mo	96.90602	9.58%
^{98}Mo	97.90540	24.29%
^{100}Mo	99.90748	9.74%

These seven isotopes give 21 pairs of isotopes with which the mass dependence of the isotope separation can be tested. These pairs are given in Table S3, along with their neutron and mass differences.

Table S3 – Mass differences between pairs of Mo isotopes

Neutron Difference	Isotope Pair	Mass Difference (Da)
1	(94,95), (95,96), (96,97), (97,98)	1.00075, 0.99884, 1.00134, 0.99938
2	(92,94), (94,96), (95,97), (96,98), (98,100)	1.99828, 1.99959, 2.00018, 2.00072, 2.00208
3	(92,95), (94,97), (95,98), (97,100)	2.99903, 3.00093, 2.99956, 3.00146
4	(92,96), (94,98), (96,100)	3.99787, 4.00031, 4.00280
5	(92,97), (95,100)	4.99921, 5.00164
6	(92,98), (94,100)	5.99859, 6.00239
8	(92,100)	8.00067

4.2. Mass Dependence Results: The centrifuge experiment chosen to test the mass dependence was the first tube of the 0.1 m Na_2MoO_4 72 hour run at 40°C since this produced a large separation and the Na_2MoO_4 was of very high purity, as given by its trace metal analysis in its certificate of analysis. Table S4 gives the measured selectivities for each pair of isotopes. The selectivity is defined as follows, where square brackets indicate concentrations:

$$\alpha = \frac{([M_1]/[M_2])_{Inner\ Radius}}{([M_1]/[M_2])_{Outer\ Radius}}$$

In agreement with the theory of Sections S2 and S3, the isotope separation factor was found to be an exponential function of the mass difference between the isotopes to very high accuracy. The coefficient of determination was 0.99999953 for $\ln(\alpha)$ being a linear function of ΔM. As can be seen in Figures S1b and S1d, the MC-ICPMS precision was mostly able to discern the slight selectivity variations between isotopes of one neutron difference, owing to their slightly different mass differences in Table S3 due to nuclear binding energies. Assuming the mass dependence of the separation to be exactly in-line with theory, an upper-bound error on the selectivity measurement in this case can be given as ±0.00015.

Moreover, the results show that the isotope separation depends only on the isotope mass difference ΔM, and not the relative mass difference $\Delta M/f(M_1M_2)$, unlike chemical exchange, gas diffusion, thermal diffusion, or distillation. Hence, liquid solution centrifugation is equally effective for both light and heavy elements on a per-neutron basis, whether this be ^{6}Li and ^{7}Li or ^{207}Pb and ^{208}Pb.

Table S4 – Measured selectivities of pairs of Mo isotopes after 72 hours centrifugation in 0.1 m Na_2MoO_4 at 40oC

Isotope	92	94	95	96	97	98	100
92	1	1.103958	1.160020	1.218756	1.280595	1.345458	1.485320
94		1	1.050783	1.103988	1.160004	1.218759	1.345450
95			1	1.050633	1.103942	1.159858	1.280426
96				1	1.050740	1.103960	1.218718
97					1	1.050651	1.159867
98						1	1.103951
100							1

Figure S1 shows plots of the isotope selectivity (Table S4) and log selectivity versus the isotope mass difference (Table S3), as well as regression lines for the data in red:

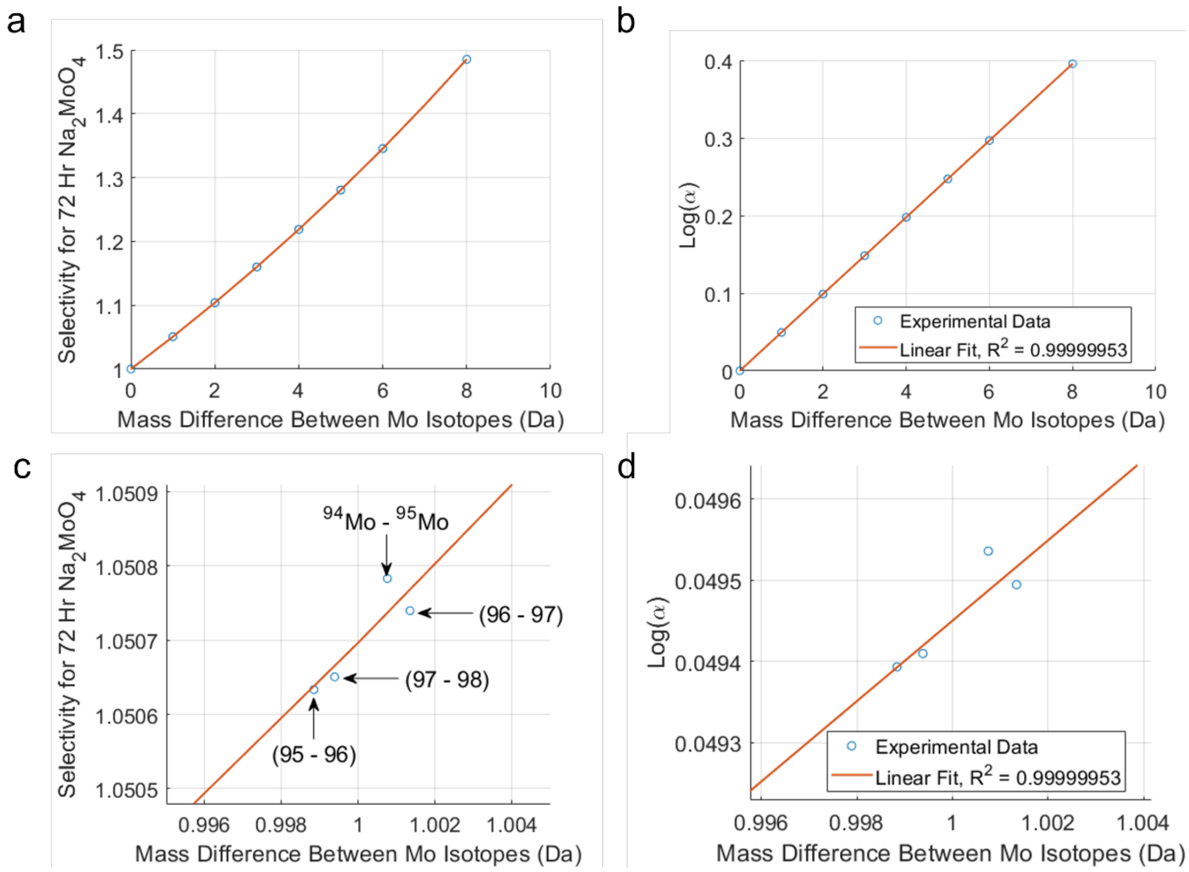

Figure S1 - Selectivity vs Mass Difference for Mo in Na_2MoO_4. (c) and (d) are zoomed in versions (~1000 x) of (a) and (b), respectively, around 1 Da. (a-d) Experimental data (blue circles) and lines of best fit (red) are plotted together. The exceptional agreement indicates an MC-ICPMS precision of better than one part in 10^4.

Section S5. Selectivity Data

5.1. Measurements and Errors: As described in Section 1, two Nu Sapphire MC-ICPMS instruments were used for all isotopic measurements apart from H and O. The typical per mil errors for these measurements, given as 2x the measurement standard deviation (SD), were:

Table S5 – Typical error in the measurements of isotopic pairs for different elements

Isotope Ratio (δ)	$^{6}Li/^{7}Li$	$^{40}Ca/^{48}Ca$	$^{95}Mo/^{98}Mo$	$^{16}O/^{18}O$	H/D	$^{39}K/^{41}K$
2 SD	0.65	0.30	0.06	0.15	0.7	0.06

A 0.5 per mil error corresponds to α ±0.0005 selectivity error.

The accuracy in table S5 corresponds to 2SD (standard deviation), Such 2SD is already after the average of three measurements. This corresponds to 1 SD of 0.33‰, 0.02‰, 0.01‰, 0.04‰, 0.35‰, and 0.015‰ per neutron difference for $^{6}Li/^{7}Li$, $^{40}Ca/^{48}Ca$, $^{95}Mo/^{98}Mo$, $^{16}O/^{18}O$, H/D, and $^{39}K/^{41}K$, respectively. As collecting samples from the top and the bottom are independent, 1SD in α is 0.02‰ + 0.02‰ = 0.04‰ per neutron for $^{40/48}Ca$, and the absolute uncertainty in α is 0.04‰× α per neutron. Here we use $\Delta(x/y)/(x/y) = \Delta x/x + \Delta y/y$, where Δ indicates uncertainty.

In addition to the uncertainty in measuring δ in samples, there is also uncertainty in obtaining the sample from the top and the bottom of the tube, which arises from factors such as the inexact position at the top and the bottom of the tube, and disturbances during the transfer of the sample out of the centrifuge.

In our experience, the position of getting a sample may vary by 2 mm with disturbances considered. Take $^{40/48}Ca$ at 24 h as an example, this will cause an uncertainty in isotope ratio of 0.0030 per neutron at bottom and 0.0013 per neutron at top based on Fig. R1 (originally Fig. 2b) below. As collecting samples from the top and the bottom are independent, the relative uncertainty in α is (0.0030/0.985)+(0.0013/1.011) = 0.0043 per neutron at 24 h, and the absolute uncertainty in α is 0.0043*(1.011/0.985) =0.0044 per neutron. Here we use $\Delta(x/y)/(x/y) = \Delta x/x + \Delta y/y$, where Δ indicates uncertainty. It should be noted that such uncertainty only causes α to decrease, but not possibly increase, since the theoretical bottom and the top should be locations with largest and smallest isotope ratios ([heavy isotope] / [light isotope]), respectively.

As uncertainties in 1) isotope measurements and 2) getting samples are independent, the overall uncertainty is calculated as follows. Take $^{40}Ca/^{48}Ca$ at 24 hrs as an example, the lower uncertainty is $((0.02‰)^2 + 0^2)^{1/2}$ = 0.02‰ per neutron, or 0.15‰ for $^{40}Ca/^{48}Ca$. The upper uncertainty is $((0.02‰)^2 + (0.0044)^2)^{1/2}$ = 4.4‰ per neutron, or is $((0.15‰)^2 + (0.0044 \times 8)^2)^{1/2}$ = 35‰ for $^{40}Ca/^{48}Ca$. The calculated uncertainties for all samples in Fig. 3d are updated as Table S6.

Table S6 – Total estimated upper and lower selectivity uncertainties for different isotope pairs and centrifugation times

Lower / upper uncertainty in α (**10^{-3}**)	$^{6}Li/^{7}Li$	$^{40}Ca/^{48}Ca$	$^{95}Mo/^{98}Mo$	$^{16}O/^{18}O$	H/D
6 h	0.66 / 3.6	0.30 / 28	0.06 / 11		
24 h	0.67 / 4.5	0.31 / 35	0.06 / 14	0.17 / 9	0.73 / 4.6
72 h	0.68 / 4.7	0.32 / 37	0.06 / 15	0.17 / 10	0.75 / 4.8
Lower / upper uncertainty	$^{6}Li/^{7}Li$	$^{40}Ca/^{48}Ca$	$^{95}Mo/^{98}Mo$	$^{16}O/^{18}O$	H/D

in α **per neutron (10^{-3})**					
6 h	0.66 / 3.6	0.04 / 3.5	0.02 / 3.6		
24 h	0.67 / 4.5	0.04 / 4.4	0.02 / 4.5	0.09 / 4.6	0.73 / 4.6
72 h	0.68 / 4.7	0.04 / 4.6	0.02 / 4.7	0.09 / 4.8	0.75 / 4.8

Similarly, the uncertainties of α at different positions can be obtained for Fig. 3c, which are 0.01 or less. For Fig. 3b, the uncertainties are listed in Table S7. The calculated uncertainties are consistent with observed fluctuation in Tables S8-13 below, which are mostly within 1-4%.

Table S7 - Upper and lower selectivity uncertainties for the Ca isotope presented in Fig. 3b

Lower / upper uncertainty for $^{40/48}$Ca (10^{-3})	α	α_{inner}	α_{outer}
6 h	0.30 / 28	0.15 / 8	20 / 0.15
24 h	0.30 / 35	0.15 / 10	25 / 0.15
72 h	0.30 / 37	0.15 / 11	26 / 0.15

Fig. 3a regards concentration, where the uncertainties can be estimated in 1) instrumental accuracy and 2) sample preparation through the same procedure above. The results are as follows. At radius of 12 cm, the concentrations are 0.27 (+0.04, -0.02) M for CaS_2O_3, 0.207 (+0.02, -0.01) M for $Ca(NO_3)_2$, and 0.16 (+0.02, -0.01) M for $CaCl_2$. At radius of 6.3 cm, the concentrations are 0.026 ± 0.006 M for CaS_2O_3, 0.046 ± 0.005 M for $Ca(NO_3)_2$, and 0.052 ± 0.004 M for $CaCl_2$.

5.2. Aqueous Experiments: The results from all aqueous experiments are given in Tables S8-S13 for Ca, Mo, H, O, Li, and K respectively. Values for the inner selectivity, outer selectivity, and total selectivity are given, with the inner and outer selectivities defined below. 1 m salt solution = 1 mol kg^{-1} water.

$$\alpha_{inner} = \frac{([M_1]/[M_2])_{Inner\ Radius}}{([M_1]/[M_2])_{Inital\ Solution}}$$

$$\alpha_{outer} = \frac{([M_1]/[M_2])_{Outer\ Radius}}{([M_1]/[M_2])_{Initial\ Solution}}$$

$$Inner\ Percentage = \log_{\alpha}(\alpha_{inner})$$

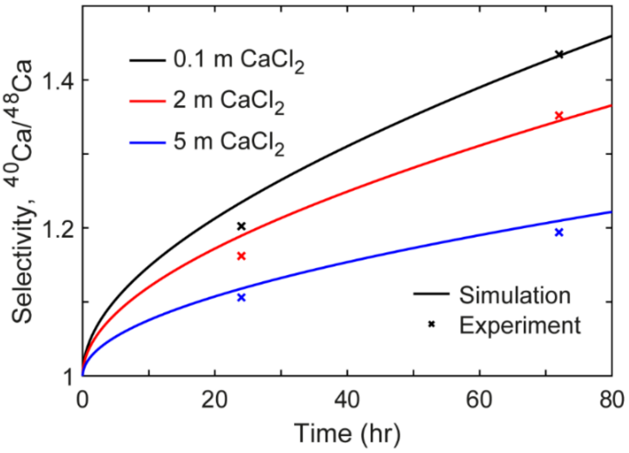

Figure S2 - The 24- and 72-hour selectivities of 3 concentrations of $CaCl_2$. Higher concentrations give lower transient results due the lower ionic diffusivities. The larger deviations from the simulated curves after 24 hours are suspected to be caused by remixing and finite sample collection.

Table S8 ($^{40}Ca/^{48}Ca$)

Sample	Time (Hours)	Inner Selectivity	Outer Selectivity	Inner Percentage	Total Selectivity
0.1 m $CaCl_2$, 1	**72**	1.2312	0.8583	57.6%	1.4345
0.1 m $CaCl_2$, 2	**72**	1.2167	0.9148*	68.8%	1.3300
0.1 m $Ca(NO_3)_2$, 1	**72**	1.2165	0.8726	59.0%	1.3941
0.1 m $Ca(NO_3)_2$, 2	**72**	1.2316	0.8734	60.6%	1.4101
0.1 m CaS_2O_3, 1	**72**	1.2267	0.9025	66.6%	1.3592
0.1 m CaS_2O_3, 2	**72**	1.2678	0.9056	70.5%	1.4000
2 m $CaCl_2$, 1	**72**	1.1616	0.8660	51.0%	1.3413
2 m $CaCl_2$, 2	**72**	1.1601	0.8580	49.2%	1.3521
2 m $Ca(NO_3)_2$, 1	**72**	1.1786	0.8776	55.7%	1.3430
2 m $Ca(NO_3)_2$, 2	**72**	1.1731	0.8630	52.1%	1.3583
1 m CaS_2O_3, 1	**72**	1.2140	0.8895	62.4%	1.3648
1 m CaS_2O_3, 2	**72**	1.2323	0.8790	61.8%	1.4019
5 m $CaCl_2$, 1	**72**	1.0878	0.9109	47.4%	1.1942
5 m $CaCl_2$, 2	**72**	1.0850	0.9124	47.1%	1.1892
5 m $Ca(NO_3)_2$, 1	**72**	1.0912	0.9211	51.5%	1.1847
5 m $Ca(NO_3)_2$, 2	**72**	1.0880	0.9196	50.2%	1.1831
0.1 m $CaCl_2$, 1	**24**	1.0947	0.9189	51.7%	1.1913
0.1 m $CaCl_2$, 2	**24**	1.0931	0.9092	48.3%	1.2023
0.1 m $Ca(NO_3)_2$, 1	**24**	1.0923	0.9129	49.2%	1.1965
0.1 m $Ca(NO_3)_2$, 2	**24**	1.0974	0.9137	50.7%	1.2011
0.1 m CaS_2O_3, 1	**24**	1.1097	0.9175	54.7%	1.2095
0.1 m CaS_2O_3, 2	**24**	1.1067	0.9283	57.7%	1.1922
2 m $CaCl_2$, 1	**24**	1.0678	0.9189	43.7%	1.1620
2 m $CaCl_2$, 2	**24**	-	0.9112	-	-
5 m $CaCl_2$, 1	**24**	1.0412	0.9417	40.2%	1.1057
5 m $CaCl_2$, 2	**24**	1.0378	0.9569	45.7%	1.0845
0.1 m CaS_2O_3, 1	**6**	1.0317	0.9613	42.9%	1.0754
0.1 m CaS_2O_3, 2	**6**	1.0205	0.9594	32.9%	1.0637

Table S9 ($^{92}Mo/^{100}Mo$)

Sample	Time (Hours)	Inner Selectivity	Outer Selectivity	Inner Percentage	Total Selectivity
0.1 m Na_2MoO_4, 1	**6**	1.0341	0.9505	39.8%	1.0880
0.1 m Na_2MoO_4, 2	**6**	1.0331	0.9526	39.6%	1.0856
0.1 m Na_2MoO_4, 1	**24**	1.1227	0.9027	53.1%	1.2437
0.1 m Na_2MoO_4, 2	**24**	1.1222	0.9011	52.5%	1.2454
0.1 m Na_2MoO_4, 1	**72**	1.2976	0.8738	65.9%	1.4850
0.1 m Na_2MoO_4, 2	**72**	1.2870	0.8756	65.5%	1.4698

Table S10 ($^{1}H/^{2}H$)

Sample	Time (Hours)	Inner Selectivity	Outer Selectivity	Inner Percentage	Total Selectivity
Water (0.5 m LiCl), 1	**24**	1.0198	0.9789	48.0%	1.0417
Water (0.5 m LiCl), 2	**24**	1.0190	0.9780	45.7%	1.0420
Water (0.5 m LiCl), 3	**24**	1.0191	0.9772	45.0%	1.0429
Water (0.5 m LiCl), 1	**72**	1.0301	0.9654	45.7%	1.0670
Water (0.5 m LiCl), 2	**72**	1.0297	0.9662	46.0%	1.0657

Table S11 ($^{16}O/^{18}O$)

Sample	Time (Hours)	Inner Selectivity	Outer Selectivity	Inner Percentage	Total Selectivity
Water (0.5 m LiCl), 1	**24**	1.0388	0.9552	45.4%	1.0875
Water (0.5 m LiCl), 2	**24**	1.0382	0.9541	44.4%	1.0882
Water (0.5 m LiCl), 3	**24**	1.0380	0.9539	44.2%	1.0881
Water (0.5 m LiCl), 1	**72**	1.0586	0.9331	45.2%	1.1342
Water (0.5 m LiCl), 2	**72**	1.0588	0.9341	46.6%	1.1335

Table S12 ($^{6}Li/^{7}Li$ Isotopes)

Sample	Time (Hours)	Inner Selectivity (α_{inner})	Outer Selectivity (α_{outer})	Inner Percentage	Total Selectivity (α)
0.1 m Li_2SO_4, 1	**72**	1.0274	0.9765	53.3%	1.0520
0.1 m Li_2SO_4, 2	**72**	1.0272	0.9768	53.4%	1.0515
1 m LiCl, 1*	**72**	1.0241	0.9804	54.5%	1.0447
1 m LiCl, 2	**72**	1.0252	0.9751	49.7%	1.0514
1 m LiBr, 1	**72**	1.0290	0.9771	55.3%	1.0531
1 m LiBr, 2	**72**	1.0290	0.9772	55.3%	1.0531
1 m LiI, 1	**72**	1.0321	0.9789	59.6%	1.0544
1 m LiI, 2	**72**	1.0316	0.9800	60.7%	1.0526
1 m Li_2MoO_4, 1	**72**	1.0188	0.9800	48.0%	1.0396
1 m Li_2MoO_4, 2	**72**	1.0208	0.9806	51.2%	1.0410
2 m LiOH, 1	**72**	1.0208	0.9761	46.0%	1.0458
2 m LiOH, 2	**72**	1.0206	0.9752	44.8%	1.0466
5 m LiCl, 1	**72**	1.0198	0.9752	43.8%	1.0458

5 m LiCl, 2	**72**	1.0204	0.9744	43.9%	1.0471
0.1 m Li_2SO_4, 1	**24**	1.0129	0.9840	44.2%	1.0294
0.1 m Li_2SO_4, 2	**24**	1.0130	0.9842	44.7%	1.0293
0.1 m Li_2MoO_4, 1	**24**	1.0134	0.9863	49.1%	1.0275
0.1 m Li_2MoO_4, 2	**24**	1.0135	0.9854	47.7%	1.0285
0.1 m $Li_2C_2O_4$,1	**24**	1.0125	0.9843	43.9%	1.0287
0.1 m $Li_2C_2O_4$, 2	**24**	1.0122	0.9853	45.0%	1.0273
0.1 m LiOH, 1	**24**	1.0085	0.9879	40.7%	1.0210
0.1 m LiOH, 2	**24**	1.0086	0.9888	43.5%	1.0199
0.5 m Li_2SO_4, 1	**24**	1.0112	0.9842	40.9%	1.0276
0.5 m Li_2SO_4, 2	**24**	1.0112	0.9845	41.7%	1.0271
0.5 m $LiNO_3$, 1	**24**	1.0123	0.9832	42.0%	1.0295
0.5 m $LiNO_3$, 2	**24**	1.0122	0.9823	40.5%	1.0304
2 m LiOH, 1	**24**	1.0107	0.9878	46.6%	1.0231
2 m LiOH, 2	**24**	1.0106	0.9873	45.2%	1.0236
20% Mass LiBr, 1	**24**	1.0128	0.9863	48.1%	1.0268
20% Mass LiBr, 2	**24**	1.0127	0.9854	46.4%	1.0276
5 m LiCl, 1	**24**	1.0104	0.9870	44.2%	1.0237
5 m LiCl, 2	**24**	1.0106	0.9869	44.5%	1.0240
10 m LiCl, 1	**24**	1.0062	0.9905	39.2%	1.0159
10 m LiCl, 2	**24**	1.0060	0.9905	38.4%	1.0157
0.1 m Li_2SO_4, 1	**6**	-	-	-	1.0107
0.1 m Li_2SO_4, 2	**6**	-	-	-	1.0108

*It is suspected that accidental knocking of the centrifuged tube during collection induced convection and reduced the results.

Table S13 ($^{39}K/^{41}K$)

Sample	Time (Hours)	Inner Selectivity	Outer Selectivity	Inner Percentage	Total Selectivity
0.1 m K_2SO_4, 1	**24**	1.0424	0.9653	54.0%	1.0799
0.1 m K_2SO_4, 2	**24**	1.0394	0.9660	52.8%	1.0760
0.5 m K_2MoO_4, 1	**72**	1.0849	0.9657	70.0%	1.1234
0.5 m K_2MoO_4, 2	**72**	1.0953	0.9653	72.0%	1.1347

5.3. Non-aqueous Experiments: Results from all non-aqueous experiments are given in Table S14.

Table S14 – Selectivity results from experiments involving non-aqueous solutions

Sample	Solvent	Time (Hours)	Inner Selectivity	Outer Selectivity	Total Selectivity
0.1 m LiBr, 1	**Propylene Carbonate**	**24**	-	-	1.0054
0.1 m LiBr, 2	**Propylene Carbonate**	**24**	-	-	1.0054
0.1 m LiCl, 1	**Dimethyl Sulfoxide**	**24**	-	-	1.0056

0.1 m LiCl, 2	**Dimethyl Sulfoxide**	**24**	-	-	1.0050
0.1 m $LiPF_6$, 1	**Propylene Carbonate**	**24**	-	-	1.0048
0.1 m $LiPF_6$, 2	**Propylene Carbonate**	**24**	-	-	1.0050
0.1 m $LiBF_4$, 1	**Propylene Carbonate**	**24**	-	-	1.0045
0.1 m $LiBF_4$, 2	**Propylene Carbonate**	**24**	-	-	1.0046
0.2 m $Ca(NO_3)_2{\cdot}4H_2O$, 1	**Triethyl Phosphate**	**24**	1.0357	0.9715	1.0661
0.2 m $Ca(NO_3)_2{\cdot}4H_2O$, 2	**Triethyl Phosphate**	**24**	1.0451	-	-
0.2 m $Ca(NO_3)_2$, 1	**Triethyl Phosphate**	**24**	1.0484	0.9722	1.0784
0.2 m $Ca(NO_3)_2$, 2	**Triethyl Phosphate**	**24**	1.0556	0.9832	1.0736

5.4. Cascade Experiments: The results from the 3-stage 72-hour $CaCl_2$ cascade are given in Table S15. All selectivity values are given with respect to the initial (natural) $^{40}Ca/^{48}Ca$ ratio.

Two independent cascades were run with the first number of each pair of values being from the first cascade and the second being from the second cascade. The table entry above each cell shows the selectivity of the solution at the beginning of the centrifuge experiments.

Table S15 – Selectivities after each stage for the 3-stages for 72 hour centrifugation

<table>
<tr><td>Stage</td><td colspan="6">Initial: 1.000</td></tr>
<tr><td>1</td><td colspan="3">Top/Inner: 1.152, 1.172</td><td colspan="3">Bottom/Outer: 0.863, 0.871</td></tr>
<tr><td>2</td><td colspan="2">Top: 1.327, 1.376</td><td>Bottom: 1.015, 1.018</td><td>Top: 1.032, 1.035</td><td colspan="2">Bottom: 0.764, 0.762</td></tr>
<tr><td>3</td><td>Top: 1.611, -</td><td>Bottom: 1.213, 1.240</td><td>-</td><td>-</td><td>Top: 0.928, 0.927</td><td>Bottom: 0.662, 0.670</td></tr>
</table>

Section S6. Concentration Data

6.1. Measurements and Errors: Concentration measurements were made using the same Nu Sapphire MC-ICPMS instruments as were used for the isotopic measurements. Concentration measurements used the most abundant isotope - ^{7}Li for lithium, ^{40}Ca for calcium etc. All concentration measurements used the initial solution before centrifugation as the reference solution. For example, when measuring the ^{7}Li concentration in LiCl at the top and bottom of the centrifuge tube after an experiment, these concentrations used the initial solution of LiCl before centrifugation as the reference. This eliminated errors in the concentration measurement which are due to the presence of different counterions or other impurities.

The combined error in the concentration measurements given is estimated at ±6-7%. This comes from the combined errors of: Sample mass measurement (<1% as 0.1 mg precision used and samples were ~25-75 mg), weighing scales used for water dilution (~2% as 1 mg precision and used down to 50-100 mg), diluting process (<1%), and actual measurement (2-3%). Such error does not affect errors in the isotope ratio, as all errors above cancel out for two isotopes. Therefore, the errors in isotope ratio measurements are still the same as those discussed in section S5.1.

Tables S16 and S17 give the measured concentration results for Ca and Li, respectively.

Table S16 - Ca concentration results after centrifugation

Sample	Time (Hours)	Inner Concentration	Outer Concentration	Isotope Selectivity
0.1 m $CaCl_2$, 1	**24**	0.0518 m	0.160 m	1.1913
0.1 m $CaCl_2$, 2	**24**	0.0514 m	0.121 m	1.2023
0.1 m $Ca(NO_3)_2$, 1	**24**	0.0466 m	0.191 m	1.1965
0.1 m $Ca(NO_3)_2$, 2	**24**	0.0462 m	0.203 m	1.2011
0.1 m CaS_2O_3, 1	**24**	0.0263 m	0.288 m	1.2095
0.1 m CaS_2O_3, 2	**24**	0.0260 m	0.260 m	1.1922

Table S17 – Li concentration results after centrifugation

Sample	Time (Hours)	Inner Concentration	Outer Concentration	Isotope Selectivity
1 m LiCl, 1	**72**	0.793 m	-	1.0447
1 m LiCl, 2	**72**	0.788 m	1.297 m	1.0514
1 m LiBr, 1	**72**	0.468 m	1.812 m	1.0531
1 m LiBr, 2	**72**	0.455 m	1.756 m	1.0531
1 m LiI, 1	**72**	0.273 m	2.000 m	1.0544
1 m LiI, 2	**72**	0.279 m	2.058 m	1.0526
1 m Li_2MoO_4, 1 (2 m Li)	**72**	1.144 m	3.865 m	1.0396
1 m Li_2MoO_4, 2	**72**	1.034 m	-	1.0410
2 m LiOH, 1	**72**	1.513 m	2.566 m	1.0458
2 m LiOH, 2	**72**	1.358 m	2.496 m	1.0466
5 m LiCl, 1	**72**	4.818 m	5.251 m	1.0458
5 m LiCl, 2	**72**	4.818 m	5.140 m	1.0471
0.1 m LiTFSI, 1	**24**	0.0388 m	0.305 m	-
0.1 m LiTFSI, 2	**24**	0.0474 m	0.295 m	-
0.1 m Li_2MoO_4, 1	**24**	0.0937 m	0.408 m	1.0275
0.1 m Li_2MoO_4, 2	**24**	0.0980 m	0.431 m	1.0285
0.1 m $Li_2C_2O_4$,1	**24**	0.1365 m	0.301 m	1.0287
0.1 m $Li_2C_2O_4$, 2	**24**	0.1372 m	0.279 m	1.0273
0.1 m LiOH, 1	**24**	0.0844 m	0.113 m	1.0210
0.1 m LiOH, 2	**24**	0.0881 m	0.117 m	1.0199
0.5 m Li_2SO_4, 1	**24**	0.708 m	1.468 m	1.0276
0.5 m Li_2SO_4, 2	**24**	0.633 m	1.522 m	1.0271
0.5 m $LiNO_3$, 1	**24**	0.395 m	0.614 m	1.0295
0.5 m $LiNO_3$, 2	**24**	0.387 m	0.676 m	1.0304
1 m LiOH, 1	**24**	0.828 m	1.176 m	-
1 m LiOH, 2	**24**	0.875 m	1.198 m	-
2 m LiOH, 1	**24**	1.900 m	2.625 m	1.0231
2 m LiOH, 2	**24**	1.944 m	2.482 m	1.0236
5 m LiCl, 1*	**24**	4.701 m	4.541 m	1.0237
5 m LiCl, 2*	**24**	4.681 m	4.740 m	1.0240
10 m LiCl, 1*	**24**	9.755 m	9.916 m	1.0159
10 m LiCl, 2*	**24**	9.412 m	9.635 m	1.0157

*It is thought that additional errors were introduce as a result of the 5 m and 10 m solution references partially evaporating and thereby increasing their concentration and artificially lowering the measured sample values. The magnitude of this additional error is estimated to be at most ±5%.

Section S7. Continuous Process Considerations

7.1. Design Considerations:

All experiments performed in this study used a biomedical ultracentrifuge and a swinging-bucket rotor (Beckman Optima XPN-100 Ultracentrifuge and a SW 60 Ti rotor). This configuration is for demonstrating the concept. In practical continuous production, the principles of modern gas centrifuges could be used to create an optimized design. These principles are:

1. An axially symmetric cylindrical centrifuge rotating in a vacuum on a needle bearing and supported by magnetic bearings
2. The flow of solution being continuous in and out of the centrifuge, such that it does not ever deviate from its working rotational speed to accelerate or slow down
3. The use of the countercurrent centrifuge method to multiply the separation factor within each stage. We believe that this flow can be induced and controlled within the centrifuge using a variety of methods including thermal gradients, conical/tapered rotors, or by the mechanism of feed introduction and product/waste removal (*11*). This requires further analysis and will be incorporated in future research
4. A broad cascade of centrifuges, whereby the product of one centrifuge becomes the feed of another in succession, leading to the final overall enrichment

7.2. Countercurrent Centrifuge Model:

A countercurrent centrifuge was modeled for isotopes of water (HDO and $H_2{}^{18}O$ inside $H_2{}^{16}O$) according to Eqn. S5.

$$\vec{J}_i = -D_i \nabla c_i + D_i \frac{\omega^2 \vec{r}}{RT} c_i M_i (1 - \bar{v}_i \rho_{soln}) + c_i \vec{\boldsymbol{V}} \quad with \quad \frac{\partial c_i}{\partial t} = -\nabla \cdot (\vec{J}_i) \qquad \text{(S5)}$$

The third term on the right represents advection, where $\vec{\boldsymbol{V}}$ is the velocity field of the flow. For a countercurrent centrifuge this is modeled to be a closed-loop flow contained within a thin stream along the boundaries of the centrifuge. Eqn. S5 was solved numerically in the axial and radial directions with time.

Figure S3 shows the modeling results upon reaching equilibrium. The flow direction was chosen to be counter clockwise so that the heavier isotopes would concentrate at the top of the centrifuge. The modeled system had an inner and outer radius of 5 cm and 10 cm, respectively, and an angular velocity of 50 kRPM. These are both a 16.7% reduction compared to the centrifuge used in this study, and therefore a 30.6% reduction in peripheral speed and 51.8% reduction in wall stress and equilibrium selectivity (under half). Even in this case, the equilibrium separation factor along the axial direction for a 60 cm tall centrifuge is >1.20 per neutron mass difference.

Fig. S3a shows the equilibrium distribution of ^{18}O throughout the centrifuge. The thin stream of counter-clockwise flow along the boundaries has the effect of concentrating the heavier isotope at the top outer-radii corner. The thin stream travels upwards at the outer radii and downwards at the inner radii. Steep concentration gradients are developed along the axial direction as shown in Fig. S3b, leading to large separation factors in that direction. In the radial direction, typical separations occur as the result of the centrifugal forces (Fig. S3c).

The radius and height of the centrifuge used for simulations in Figure S3 is based on the conventional Zippe-type centrifuge, which has been used for the countercurrent configuration in gas centrifugation. The design is different from the lab-scale swing-bucket centrifuge we used in Figure 1.

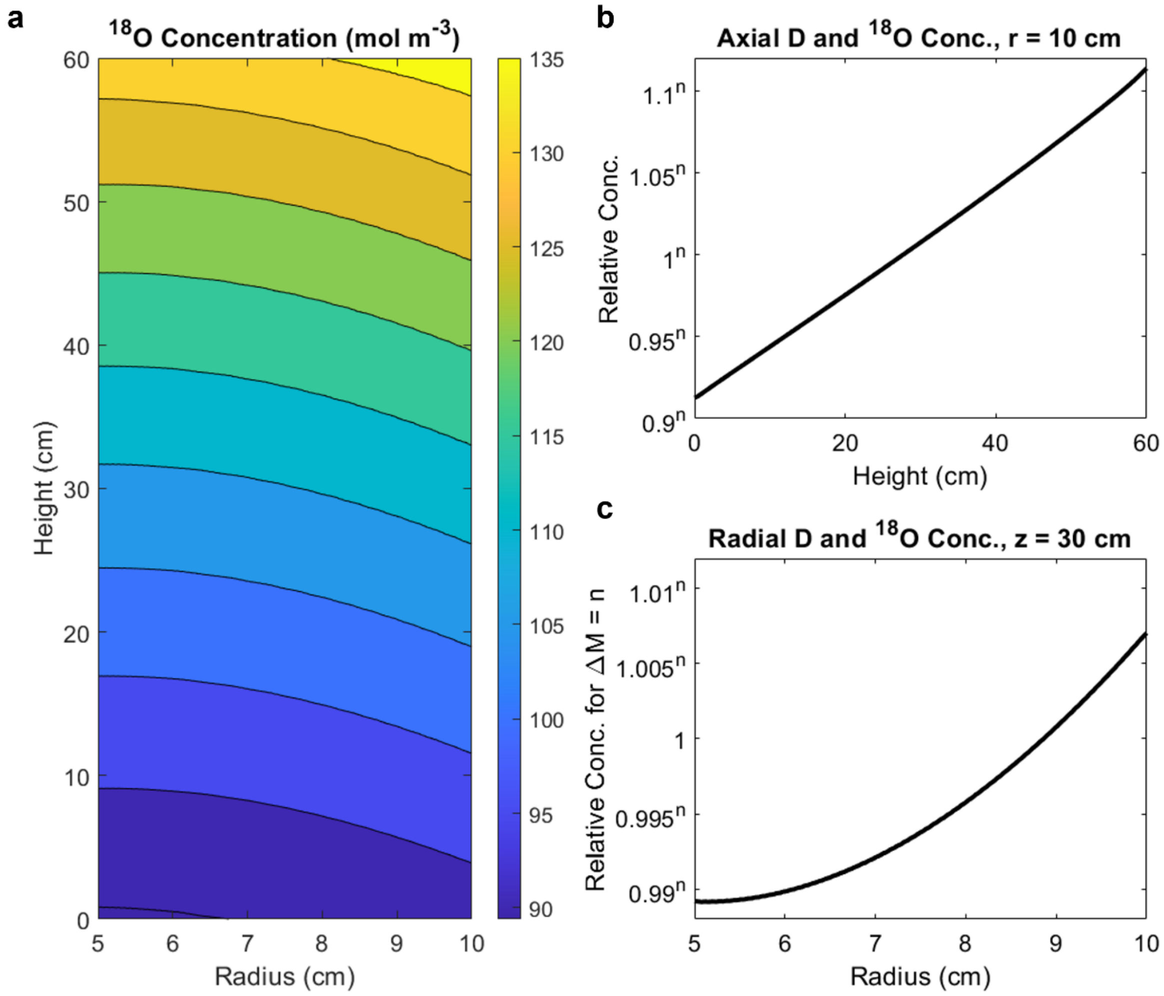

Figure S3 - The equilibrium countercurrent centrifugation modeling results. (a) The distribution of $H_2{}^{18}O$ throughout the centrifuge. (b) The relative concentration of isotope species as a function of their mass difference from $H_2{}^{16}O$ along the outer radius axial line. (E.g., ΔM = 1 from HDO). (c) The relative concentration of isotope species along the z = 30 cm horizontal centerline.

7.3 Considerations on Equilibrium Time

The liquid centrifuge is expected to have a significantly longer time to reach steady state due to the lower mass diffusivity in liquids than gases. It is believed that this can be partly remedied in two ways and that this may not ultimately be a serious consequence for a separate reason.

1. By operating the liquid centrifuge closer to the liquid boiling point than the melting point, the diffusion coefficient can be enhanced by >3-5x without any noticeable loss in the density and concentration (Fig. S4).
2. Using smaller centrifuges with the same peripheral speed. Since the equilibrium time in diffusion phenomena is proportional to the square of the length scale, by scaling down the centrifuge by a factor of two, for example, the time to equilibrium can be reduced by a factor of four. Further, since the stress induced inside geometrically similar rotating cylinders is only a function of their peripheral speed squared, smaller centrifuges can achieve the same single-

stage separation factors as larger centrifuges, since the equilibrium separation factor and bursting strengths are both functions of this peripheral speed squared.

Additionally, although the time to reach equilibrium may be several weeks or longer in a theoretical liquid centrifuge cascade, it can be noted that this only needs to occur once for each period of continuous operation, which could last years or decades in the case of gas centrifuge cascades. Therefore, the slower approach towards equilibrium has less of an effect in continuous operations than in a batch-process since it represents a much smaller proportional of the overall time.

Section S8. Throughput Analysis

The separative throughput of a centrifuge is proportional to the product of the concentration and diffusion coefficient of the target species, i.e., J ∝ c×D (*11*). The number of cascade stages in a centrifuge is inversely proportional to ln(α) ∝ ΔM. So J × ln(α) is also a meaningful parameter to evaluate the separation power of a system.

8.1. Comparison of Gas and Liquid Centrifuge:

For gases, the diffusivity can be modeled using Chapman–Enskog theory as in Eqn. S6

$$D = \frac{AT^{3/2}}{p\sigma_{12}^2\Omega}\sqrt{\frac{1}{M}} \qquad \text{(S6)}$$

where A is an empirical coefficient, T is the absolute temperature, M is the molar mass, p is the pressure, σ_{12} is the average collision diameter and Ω is a temperature-dependent collision integral. As $p = cRT$ based on the ideal gas law, c×D is a constant at a given temperature so that changes in the pressure and gas concentration do not affect this parameter (*32*). Moreover, the temperature dependence of c×D is $T^{1/2}$, which is a weak dependence. From 25 to 100 °C, c×D only increases by ~12% for a gas, as given in Figure S4a. This originates from the fact that *c* decreases at a higher temperature and the same pressure.

In contrast, for species in a liquid solution, the product c×D is itself a non-linear function of the solute concentration and can therefore be maximized for a given solute. c×D also increases more significantly in an aqueous solution than gas. As shown in Figure S4a, between 25 and 100 °C, the diffusion coefficient increases ~ 2-2.5% K^{-1} of that at 25°C, which is much stronger than gas (*25*). The solubility can also increase by 50 – 200 % compared to that at 25 °C. Hence, c×D can increase by 50-100% from 25 to 50 °C, and even 2-5x from 25 to 100 °C. The exact increase is solution-specific and can be optimized by tuning the chemical composition of solutes and solvents.

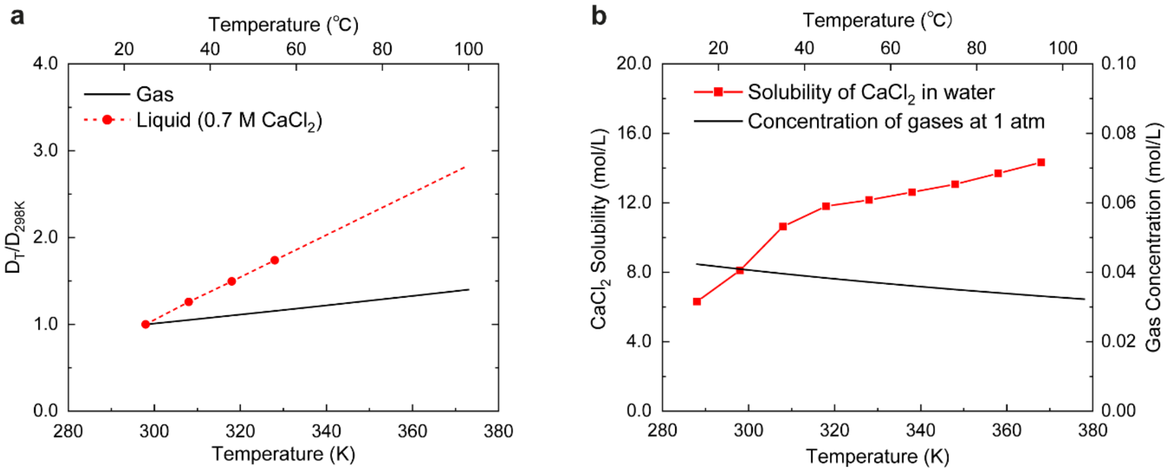

Figure S4 – **Temperature dependences.** (a) The temperature dependence of diffusivity for gas and liquid (0.7 M $CaCl_2$ aqueous solution). (b) The temperature dependence of $CaCl_2$ solubility in water and the temperature dependence of concentration for gaseous species at 1 atm.

8.2. Summary of throughput values for gas and liquid centrifuge:

Table S19 gives some values for c×D and c×D×ln(α) at two concentrations and temperatures for the some of the salts used in this study, and the flux comparison between gaseous and liquid species is provided. The term ln(α) is represented by ΔM/T, as the selectivity is an exponential function of the isotope mass difference and the inverse temperature. At steady state, the flux of liquid centrifugation is about ~1/10 of gas centrifugation at moderate temperature but this could be elevated with higher temperatures. Even at 50°C the separation for $^{40}Ca/^{48}Ca$ and $^{35}Cl/^{37}Cl$ show good throughput potential, and especially $^{1}H/^{2}H$ and $^{16}O/^{18}O$ due to the high solvent concentration. Most importantly, all 4 of these isotope pairs could be simultaneously separated using $CaCl_{2(aq)}$.

Table S18 – Concentration-diffusivity products for $UF_{6(g)}$ at various temperatures

**Gas**	**Temperature (°C)**	**c×D×10⁶ (∝ Flux) (mol m⁻¹ s⁻¹)**	**c×D×ln(α)×10⁹ (mol m⁻¹ s⁻¹)**
**UF_6**	25	61.7	**621**
	50	66.5	**617**
	75	71.2	**614**

Table S19 - Concentration-diffusivity products for numerous elements in aqueous solutions at various temperatures

**Liquid Solution**	**Temperature (°C)**	**Concentration (mol L⁻¹)**	**Diffusion Coefficient (10^9 m² s⁻¹)**	**c_i×D_i ×10^6 (∝ Flux) (mol m⁻¹ s⁻¹)**	**c_i×D_i ×ln(α)×10^9 (mol m⁻¹ s⁻¹)**
$\mathbf{Ca}Cl_{2(aq)}$	25	1.0 M	1.22	1.22	32.7
		5.0 M	0.72	3.60	96.6
	50	1.0 M	2.06	2.06	51.0
		5.0 M	1.21	6.05	150
	75	1.0 M	3.48	3.48	80.0
		5.0 M	2.07	10.35	237.9
	100	1.0 M	5.87	5.87	126.0
		5.0 M	3.49	17.45	**374**
$\mathbf{Li}Cl_{(aq)}$	25	1.0 M	0.93	0.93	3.1
		5.0 M	0.62	3.44	11.5
	50	1.0 M	1.50	1.50	4.6
		5.0 M	0.91	5.10	**15.8**
$Li\mathbf{Cl}_{(aq)}$	25	1.0 M	1.68	1.68	11.3
		5.0 M	1.05	5.25	35.2
	50	1.0 M	2.47	2.47	15.3
		5.0 M	1.54	7.70	**47.7**
$\mathbf{H_2}O$	25	110.8 M	2.32	257	863
	50	109.8 M	3.89	427	1322
	75	108.4 M	5.93	643	**1847**
$H_2\mathbf{O}$	25	55.4 M	2.32	129	863
	50	54.9 M	3.89	214	1322
	75	54.2 M	5.93	321	**1847**
$\mathbf{K_2}MoO_{4(aq)}$	25	c_{K+}=1.0 M	1.891	1.89	12.7
		c_{K+}=5.0 M	1.249	6.99	46.9
	50	1.0 M	3.039	3.04	18.8

		5.6 M	1.85	10.40	**64.4**
K_2**Mo**$O_{4(aq)}$	25	1.0 M	0.694	0.69	18.5
		5.0 M	0.407	2.04	54.7
	50	1.0 M	1.172	1.17	29.0
		5.0 M	0.688	3.44	**85.2**

Section S9. Cost Analysis

A preliminary cost analysis can be made for liquid solution centrifugation based on its similarities to the gas centrifuge method.

Available data from the Energy Information Administration (EIA) 2021 Uranium Marketing Annual Report indicates the average price of separative work unit (SWU) for uranium isotopes in a gas centrifuge is $100 from 2020-2021 (*26*). The reported cost estimates for gas centrifuge plants indicate that the power consumption is about 62 kWh/SWU, corresponding to ~$7/SWU (Table S20) (*27*). The remaining $93/SWU is attributed to the capital and operational costs which are assumed as the same for both gas and liquid centrifugation methods. Under this assumption, the main cost difference between the two methods arises from the power requirements of operation, which can be attributed to the frictional losses of the bearing and scoops, as well as keeping the vacuum (*11*). Therefore, the power costs will be proportional to the centrifugation time, and so analogous power requirements can be made. Taking calcium as an example (e.g., 5 M $CaCl_2$ water at 50°C), the centrifugation time is inversely proportional to the product of the flux of the raw materials. A direct comparison can then be made with $UF_{6(g)}$ from Table 18.

$$t_{\text{multiplier}} = \left(\frac{J_{CaCl_2}}{J_{UF_6}}\right)^{-1} = \frac{J_{UF_6}}{J_{CaCl_2}} = \frac{6.53 \times 10^{-5}\, mol^{-1} \cdot m \cdot s}{6.05 \times 10^{-6}\, mol^{-1} \cdot m \cdot s} = 10.8$$

Therefore, the centrifugation time for $^{40}Ca/^{48}Ca$ is about 10.8 times longer than for uranium for the same molar throughput, with a corresponding power cost of 10.8 times larger per mole. Since fluxes are typically expressed in molar quantities but separative work units are per kg, it is necessary to convert between them using the molar mass of the element. The unit of Molar Separative Work (MSW) is then introduced for comparison, as moles are typically used in scientific contexts. 1 MSW is defined as 1 mole of separative work, and therefore the 1 MSW = 0.238 SWU for Uranium.

The gas centrifuge costs for uranium isotopes are then $23.8 per MSW (0.238 kg/mol × $100), with $1.67 being the power costs and $22.13 being the capital/operational costs. The costs for $^{40}Ca/^{48}Ca$ separation are then (10.8×$1.67 + $22.13) = $40.17 per mole of separative work (MSW).

Larger separation factors α per stage will increase the overall production output proportionally. This will benefit isotopes pairs with a large ΔM and disadvantage those with ΔM = 1 or 2 Da comparatively. As shown in Section S4, α is exponential with ΔM, and the number of cascade stages is inversely proportional to ln(α). Hence, the separation of $^{40}Ca/^{48}Ca$ is 8/3 = 2.67 times as efficient as for $^{235}U/^{238}U$ for a given centrifuge cascade, resulting in a cost of $40.2 / 2.67 = $15.1 / MSW output.

Similar cost estimations for enriching isotopes using liquid centrifugation can be applied to other elements. The analysis does not account for the possibility of using the same salt to separate the isotopes of multiple elements, as well as those in the solvent, which would improve overall cost effectiveness.

It is acknowledged that the assumptions made in this analysis are broad and that real production costs will vary greatly, particularly as centrifuge designs will differ and that handling liquids and gases is not analogous. It is hoped that this acts simply as a first order-of-magnitude approximation for the potential costs of such a process. It particular, it is suspected that the actual power requirements for continuous liquid devices may be higher than the gas equivalent due to the greater required wall

thickness, greater cylinder and fluid mass, and great fluid viscosity, all of which contribute to power consumption.

Table S20 – Levelized SWU costs, operating centrifuge capacity (Europe and Japan) (5% cost of capital, 6.51% capital recovery factor, +0% IDC, 0% contingency). Reproduced with permission from (*27*) - G. Rothwell, Market Power in Uranium Enrichment. *Sci. Glob. Secur.* **17**, 132-154 (2009).

Firm<br>Plant	(2008$)	Urenco<br>Capenhurst	Urenco<br>Almelo	Urenco<br>Gronau	JNFL<br>Rokkasho
**Plant capacity**	t SWU/yr	**3,400**	**2,900**	**1,800**	**1,500**
Overnight cost	$M	$2,342	$2,076	$1,445	$1,095
Total capital invest cost	$M	$2,342	$2,076	$1,445	$1,095
**Capital/SWU**	$/SWU	$**44.82**	$**46.56**	$**52.21**	$**56.98**
Staff size	people	340	317	257	219
Annual fully burden salary	$k/yr	$120	$120	$120	$120
**Labor/SWU**	$/SWU	$**11.99**	$**13.10**	$**17.12**	$**20.99**
Electricity consumption	kWh/SWU	62	62	62	62
Electricity price	$/MWh	$107	$107	$107	$107
**Electricity/SWU**	$/SWU	$**6.65**	$**6.65**	$**6.65**	$**6.65**
**Materials/SWU**	$/SWU	$**6.89**	$**7.16**	$**8.03**	$**8.76**
**Annual total costs**	$M	$**239**	$**213**	$**151**	$**117**
**Levelized SWU cost**	$/SWU	$**70**	$**73**	$**84**	$**93**

Section S10: Solution Non-idealities

Debye-Hückel theory treats the solvent as only a mediator for the electrostatic interactions between the dissolved ions in a solution. Therefore, the only property of importance is the dielectric constant, ϵ_r, as this relates to the electric field strength away from a charge, as given in Eqn. S7, where all terms are defined in (*29*). Since this is a constant for a given solvent, it can be factored out. Therefore, the deviation of the thermodynamic factor away from one is inversely proportional to the dielectric constant, as in Eqn. S8.

$$ln(\gamma_i^{DH}) = \frac{-q_i^2}{8\pi\epsilon_r\epsilon_o k_B T(R_i + l_D)} \qquad (S7)$$

$$\vartheta = 1 + c\frac{\partial ln(\gamma)}{\partial c} \quad \rightarrow \quad \vartheta - 1 = \frac{1}{\epsilon_o} c \frac{\partial}{\partial c}\left[\frac{-q_i^2}{8\pi\epsilon_r k_B T(R_i + l_D)}\right] \qquad (S8)$$

It must be noted that Debye-Hückel theory and its extensions are only valid to low concentrations of <0.1 M or <0.5 M where the association of ions is not significant. Beyond this, ion association and other neglected factors become large and the predictions have not agreed with experiments (*29*). No theory yet proposed has been able to accurately quantify activity coefficients for aqueous solutions at high salt concentration, and almost no attention has been given to organic solvents.

REFERENCES AND NOTES

1. Meeting Isotope Needs and Capturing Opportunities for the Future: The 2015 Long Range Plan for the DOE-NP Isotope Progarm, NSAC Isotopes Subcommitee (U.S. Department of Energy, 2015); www.osti.gov/servlets/purl/1298983.

2. J. W. Beams, L. B. Snoddy, A. R. Kuhlthau, paper presented at the Second U.N. Conference on the Peaceful Uses of Atomic Energy, 31 October 1958.

3. L. O. Love, Electromagnetic separation of isotopes at Oak Ridge. *Science* **182**, 343–352 (1973).

4. T. Graham, On the molecular mobility of gases. *Phil. Trans. R. Soc* **153**, 385–405 (1863).

5. H. C. Urey, The thermodynamic properties of isotopic substances. *J. Chem. Soc.* **1**, 562–581 (1947).

6. V. S. Letokhov, Laser isotope separation. *Nature* **277**, 605–610 (1979).

7. R. L. Murray, K. E. Holbert, in *Nuclear Energy, Isotope Separators.* (Wiley, ed. 8, 2020), chap. 15.

8. A. N. Cheltsov, L. Y. Sosnin, V. K. Khamylov, Centrifugal enrichment of nickel isotopes and their application to the development of new technologies. *J. Radioanal. Nucl. Chem.* **299**, 981–987 (2014).

9. J. Bigeleisen, M. G. Mayer, Calculation of equilibrium constants for isotopic exchange reactions. *J. Chem. Phys.* **15**, 261–267 (1947).

10. L. Onsager, R. M. Fuoss, Irreversible processes in electrolytes. Diffusion, conductance and viscous flow in arbitrary mixtures of strong electrolytes. *J. Phys. Chem.* **36**, 2689–2778 (1932).

11. H. W. Hsu, *Separations by centrifugal phenomena* (Wiley, 1981).

12. M. O. T. Osawa, F. Esaka, S. Okayasu, Y. Iguchi, T. Hao, M. Magara, T. Mashimo., Mass-dependent isotopic fractionation of a solid tin under a strong gravitational field. *EPL* **85**, 64001 (2009).

13. M. O. T. Mashimo, X. Huang, Y. Iguchi, S. Okayasu, K. Kobayashi, E. Nakamura, Isotope separation by condensed matter centrifugation: Sedimentation of isotope atoms in Se. *J. Nucl. Sci. Technol.* **45**, 105–107 (2014).

14. M. Ono, T. Mashimo, Sedimentation process for atoms in a Bi-Sb system alloy under a strong gravitational field: A new type of diffusion of substitutional solutes. *Philos. Mag. A* **82**, 591–600 (2002).

15. X. W. X. Ge, M. Zhang, S. Seetharaman, Correlation and prediction of activity and osmotic coefficients of aqueous electrolytes at 298.15 K by the modified TCPC model. *J. Chem. Eng. Data* **52**, 538–547 (2007).

16. Y. S. N. Xin, C. J. Radke, J. M. Prausnitz, Osmotic and activity coefficients for five lithium salts in three non–aqueous solvents. *J. Chem. Thermodyn.* **132**, 83–92 (2019).

17. D. Brugge, J. L. delemos, C. Bui, The sequoyah corporation fuels release and the church rock spill: Unpublicized nuclear releases in american indian communities. *Am. J. Public Health* **97**, 1595–1600 (2007).

18. B. L. Zaret, F. J. Wackers, Nuclear cardiology. *N. Engl. J. Med.* **329**, 855–863 (1993).

19. A. A. Palko, J. S. Drury, G. M. Begun, Lithium isotope separation factors of some two-phase equilibrium systems. *J. Chem. Phys.* **64**, 1828–1837 (1976).

20. D. Zucker, J. S. Drury, Separation of calcium isotopes in an amalgam system. *J. Chem. Phys.* **41**, 1678–1681 (1964).

21. A. Rittirong, T. Yoshimoto, R. Hazama, T. Kishimoto, T. Fujii, Y. Sakuma, S. Fukutani, Y. Shibahara, A. Sunaga, Isotope separation by DC18C6 crown-ether for neutrinoless double β decay of ^{48}Ca. *J. Phys.: Conf. Ser.* **2147**, 012015 (2022).

22. W. M. Rutherford, K. W. Laughlin, Separation of calcium isotopes by liquid phase thermal diffusion. *Science* **211**, 1054–1056 (1981).

23. F. M. W. Dai, M. Paquet, J. Moureau, B. Debret, J. Siebert, Y. Gerard, Y. Zhao, Calcium isotope measurements using a collision cell (CC)-MC-ICP-MS. *Chem. Geol.* **590**, 120688 (2022).

24. H. Chen, N. J. Saunders, M. Jerram, A. N. Halliday, High-precision potassium isotopic measurements by collision cell equipped MC-ICPMS. *Chem. Geol.* **578**, 120281 (2021).

25. E. A. Hollingshead, A. R. Gordon, The differential diffusion constant of calcium chloride in aqueous solution. *J. Chem. Phys.* **9**, 152–153 (1941).

26. U. S. EIA, "*Uranium Marketing Annual Report*" (EIA, 2022).

27. G. Rothwell, Market power in uranium enrichment. *Sci. Glob. Secur.* **17**, 132–154 (2009).

28. M. Blau, R. Ganatra, M. A. Bender, ^{18}F-fluoride for bone imaging. *Semin. Nucl. Med.* **2**, 31–37 (1972).

29. J.-L. Liu, C.-L. Li, A generalized Debye-Hückel theory of electrolyte solutions. *AIP Adv.* **9**, 015214 (2019).

30. F. A. Lindemann, F. W. Aston, XLVIII. The possibility of separating isotopes. *Lond. Edinb. Dublin Philos. Mag. J. Sci.* **37**, 523–534 (1919).

31. G. J. Hooyman, *"Thermodynamics of diffusion and sedimentation" in Ultracentrifugal Analysis in Theory and Experiment*. J. W. Williams, Ed. (Academic Press, 1963), pp. 3–12.

32. M. Brown, E. G. Murphy, Measurements of the self-diffusion coefficient of uranium hexafluoride. *Trans. Faraday Soc.* **61**, 2442–2446 (1965).
